# Herds Overhead: *Nimbadon lavarackorum* (Diprotodontidae), Heavyweight Marsupial Herbivores in the Miocene Forests of Australia

**DOI:** 10.1371/journal.pone.0048213

**Published:** 2012-11-21

**Authors:** Karen H. Black, Aaron B. Camens, Michael Archer, Suzanne J. Hand

**Affiliations:** 1 School of Biological, Earth and Environmental Sciences, University of New South Wales, Sydney, New South Wales, Australia; 2 Department of Earth and Environmental Sciences, University of Adelaide, Adelaide, South Australia, Australia; Monash University, Australia

## Abstract

The marsupial family Diprotodontidae (Diprotodontia, Vombatiformes) is a group of extinct large-bodied (60–2500 kg) wombat-like herbivores that were common and geographically widespread in Cenozoic fossil deposits of Australia and New Guinea. Typically they are regarded to be gregarious, terrestrial quadrupeds and have been likened in body form among placental groups to sheep, rhinoceros and hippopotami. Arguably, one of the best represented species is the zygomaturine diprotodontid *Nimbadon lavarackorum* which is known from exceptionally well-preserved cranial and postcranial material from the middle Miocene cave deposit AL90, in the Riversleigh World Heritage Area, northwestern Queensland. Here we describe and functionally analyse the appendicular skeleton of *Nimbadon lavarackorum* and reveal a far more unique lifestyle for this plesiomorphic and smallest of diprotodontids. Striking similarities are evident between the skeleton of *Nimbadon* and that of the extant arboreal koala *Phascolarctos cinereus*, including the powerfully built forelimbs, highly mobile shoulder and elbow joints, proportionately large manus and pes (both with a semi-opposable digit I) and exceedingly large, recurved and laterally compressed claws. Combined with the unique (among australidelphians) proportionately shortened hindlimbs of *Nimbadon*, these features suggest adept climbing ability, probable suspensory behaviour, and an arboreal lifestyle. At approximately 70 kg, *Nimbadon* is the largest herbivorous mammal to have occupied the forest canopies of Australia - an ecological niche that is no longer occupied in any Australian ecosystem and one that further expands the already significant niche diversity displayed by marsupials during the Cenozoic.

## Introduction

The diprotodontian marsupial Suborder Vombatiformes is represented by only four extant species: the Koala, *Phascolarctos cinereus*, sole surviving member of the once diverse (18 species within eight genera) family Phascolarctidae (Infraorder Phascolarctomorphia); and three fossorial species of wombat (Infraorder Vombatomorphia, Vombatidae). Extinct koalas, like their modern counterpart, are interpreted to have been arboreal folivores on the basis of their relatively conservative selenodont dental morphology [1]–[3], although postcranial material is not known. In contrast, the primarily terrestrial Vombatomorphia is both taxonomically (approx. 41 genera) and ecologically diverse with six extinct families identified [4]–[6] including: Diprotodontidae (marsupial sheep, hippos and rhinos), Palorchestidae (marsupial tapirs); Thylacoleonidae (carnivorous marsupial lions); Ilariidae (marsupial anthracotheres); Wynyardiidae (dog-sized quadrupedal browsers); and Maradidae (an enigmatic monotypic family of which little is currently known).

Of these groups, the diprotodontids are taxonomically the most diverse (18 genera, 30 spp) and are common components of fossil assemblages throughout Australia and New Guinea spanning the last 25 million years. While many vombatomorphian families of large herbivores (e.g. ilariids, wynyardiids, and maradids) were extinct by early Miocene times, diprotodontids were one of the few to increase in diversity throughout the Cenozoic, appearing to benefit from the opening up of Australia's forests [3]. As such, diprotodontids are arguably the most useful mammalian group for biocorrelating Australia's otherwise undated Cenozoic fossil assemblages [5]–[9].

Diprotodontids were the largest herbivores in Australasian Cenozoic palaeocommunities until their extinction in the late Pleistocene and demonstrate a general trend of increasing body size through time [7]–[10] ranging from sheep- (e.g. *Ngapakaldia tedfordi*) and calf-sized (e.g. *Ngapakaldia bonythoni*, *Neohelos tirarensis*) forms during the late Oligocene and culminating in the 2.5 tonne Pleistocene giant *Diprotodon optatum*, the biggest marsupial known. The group is characterised by simple bilophodont molars, similar to those found in extant browsing kangaroos. While some are represented by complete crania and postcranial elements, there have been few studies of the latter (e.g., [7], [8], [11], [12]). Consequently, relatively little is understood about their palaeobiology. Nevertheless, on the basis of their high relative-abundance in fossil assemblages and large body size (with most species exceeding 200 kg in weight), diprotodontids are generally interpreted to have been gregarious terrestrial quadrupeds [3].

**Figure 1:**
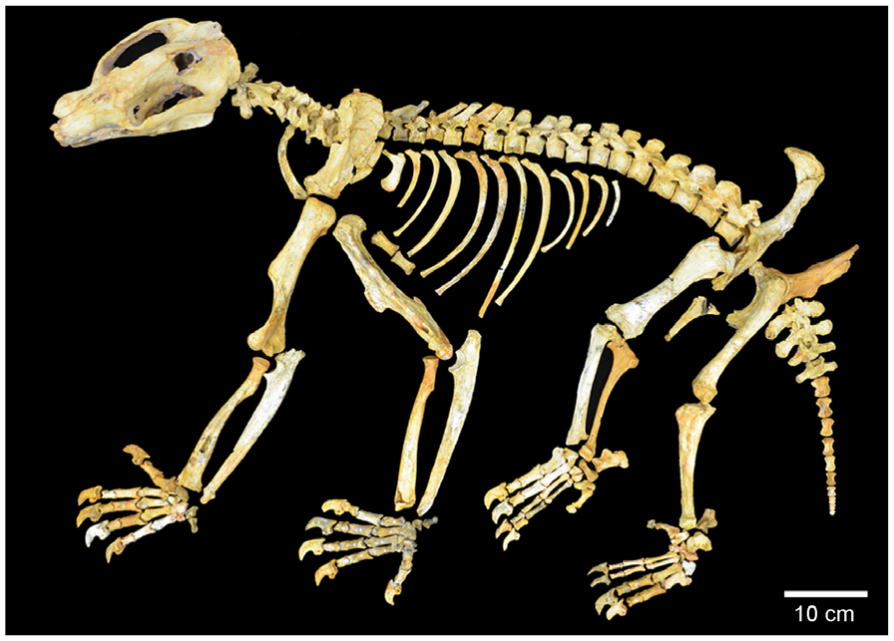
Composite *Nimbadon lavarackorum* skeleton from AL90, Riversleigh.

The middle Miocene *Nimbadon lavarackorum* [13] is among the smallest (c. 70 kg, see Text S2), most plesiomorphic [2], [14] and best represented diprotodontid species known. Exceptionally well-preserved fossils of this species (Figure 1) including more than 26 complete skulls and several articulated skeletons, ranging in age from pouch young to mature adults, have been recovered from a middle Miocene cave deposit, AL90 Site, in the Riversleigh World Heritage Area of northwestern Queensland, Australia [15], [16].

**Figure 2:**
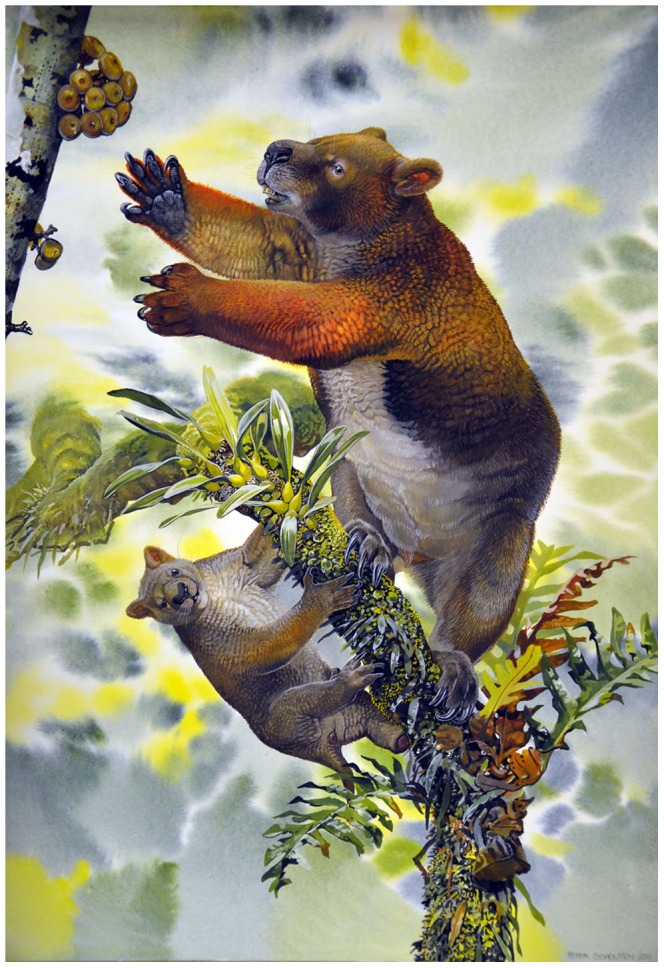
Reconstruction of *Nimbadon lavarackorum* mother and juvenile (Peter Schouten).

When first described primarily on the basis of the dentition, it was assumed that *N. lavarackorum*, like other diprotodontids, was a terrestrial quadruped although a recent analysis [17] of digital proportions and certain elements of the carpus of *N. lavarackorum* did suggest possible climbing abilities. Now, on the basis of analysis of much of the rest of the skeleton and in particular the limbs, we suggest that this species had an arboreal lifestyle (Figure 2). This makes it the largest known herbivorous mammal to occupy the forest canopies of Australia and one of the largest arboreal mammals in the world.

## Materials and Methods

*Nimbadon* specimens are registered (QM F) in the palaeontological collections of the Queensland Museum. Skeletal comparisons were made with the arboreal Koala (*Phascolarctos cinereus*), the fossorial Common Wombat (*Vombatus ursinus*), the scansorial Brushtail Possum (*Trichosurus vulpecula*) and several extinct marsupials including the diprotodontids *Ngapakaldia tedfordi*, *Ng. bonythoni*, *Neohelos tirarensis*, *Neohelos stirtoni*, *Zygomaturus trilobus, Euowenia grata*, and *Diprotodon optatum*, and the vombatomorphian Marsupial Lion (*Thylacoleo carnifex*). Comparisons were also made with the extant Malayan Sun Bear *Ursus malayanus*, a potential modern functional analogue. Specimens examined in this study are listed in Text S1 along with osteological descriptions, comparisons and functional anatomy. Reference to comparative taxa in the text is made using their generic name. *Ngapakaldia* refers to *Ngapakaldia tedfordi*, unless otherwise stated. A summary of key aspects of structure and function of the appendicular skeleton is presented below.

## Results

### Functional morphology of the forelimb

**Figure 3:**
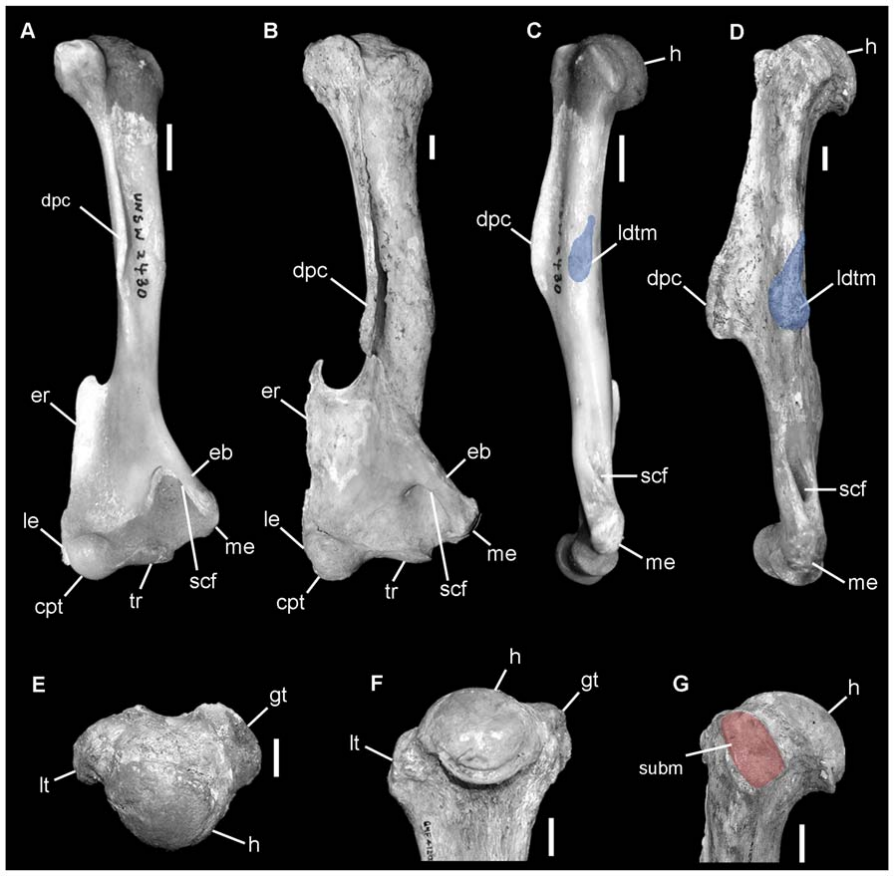
Comparison of right humerus of *Nimbadon lavarackorum* (B, D–G; QM F41202e) and *Phascolarctos cinereus* (A, C; UNSW2430). **A–B**, anterior view; **C–D**, medial view; **E**, proximal view; **F**, proximal humerus in posterior view; **G**, proximal humerus in medial view. Abbreviations: cpt, capitulum; dpc, deltopectoral crest; eb, entepicondylar bridge; er, ectepicondylar ridge; gt, greater tuberosity; h, head; ldtm, latissimus dorsi and teres major insertions (blue); le, lateral epicondyle; lt, lesser tuberosity; me, medial epicondyle; scf, supracondylar foramen; subm, subscapularis insertion on lesser tuberosity (red); tr, trochlea. Scale bars = 1 cm.

*Humerus* (Figure 3): The humerus of *Nimbadon* is strikingly similar to that of *Phascolarctos* in overall proportions and the orientation of the diaphysis. The deltopectoral crest (Figure 3A–D) is more prominent and distally extensive than in *Phascolarctos* and has a larger area for insertion of M. pectoralis suggesting greater mechanical advantage of this muscle in *Nimbadon*. The insertion areas of the latissimus dorsi and teres major muscles on the medial face of the diaphysis (Figure 3C–D) are more highly developed in *Nimbadon* than in any other taxon studied. The combined action of these muscles suggests the capacity for powerful adduction, internal rotation and retraction of the forelimb. A large triangular insertion area extending from the distal border of the greater tuberosity to the lateral superior border of the deltopectoral crest, indicates M. deltoideus pars acromialis, a main abductor and external rotator of the humerus, was similarly developed to that found in both *Phascolarctos* and *Trichosurus*.

The humeral head is broad and round and projects further proximally than the well developed humeral tuberosities (Figure 3F) indicating a highly mobile gleno-humeral joint. Significant stability of this joint is provided by well developed rotator cuff musculature indicated by: deep sulci on the proximal and lateral surfaces of the greater tuberosity for insertion of M. supraspinatus and M. infraspinatus, respectively; and a well developed muscle scar on the medial face of the lesser tuberosity for insertion of M. subscapularis (Figure 3G), which is better developed and more distinct in *Nimbadon* than in any of the other taxa examined.

The distal humerus is broad with a large, prominent and heavily pitted medial epicondyle for the origin of M. pronator teres and the carpal and digital flexors (M. flexores digitorum profundus and superficialis, and carpi radialis). It is similar in proportion to *Ngapakaldia*, slightly more laterally extensive than in *Phascolarctos* yet not as well developed as in *Vombatus*. Extending proximally and laterally from the medial epicondyle is a broad, thick entepicondylar bridge which traverses a large supracondylar foramen (Figure 3B, D). This foramen functions as a retinaculum for the median nerve and brachial artery to prevent them from slumping across the angle of the elbow during flexion. In mammals, it is associated with the ability to abduct and supinate the forearm [18]. Its large size in *Nimbadon* may indicate the forelimbs were usually held in a flexed position.

The ectepicondylar ridge is more laterally extensive in *Nimbadon* than in either *Phascolarctos*, *Vombatus* or *Trichosurus*, and is indicative of a well developed M. brachioradialis muscle (a major flexor of the forearm onto the humerus) as well as providing the site of origin for the M. extensor carpi radialis. As in *Phascolarctos* the epicondylar ridge of *Nimbadon* terminates in a well-developed hook-like process (Figure 3A–B). The lateral epicondyle (the origin of the carpal and digital extensors) is less prominent than that of *Vombatus* yet similarly developed to that of *Phascolarctos* and *Trichosurus*.

#### Elbow joint

**Figure 4:**
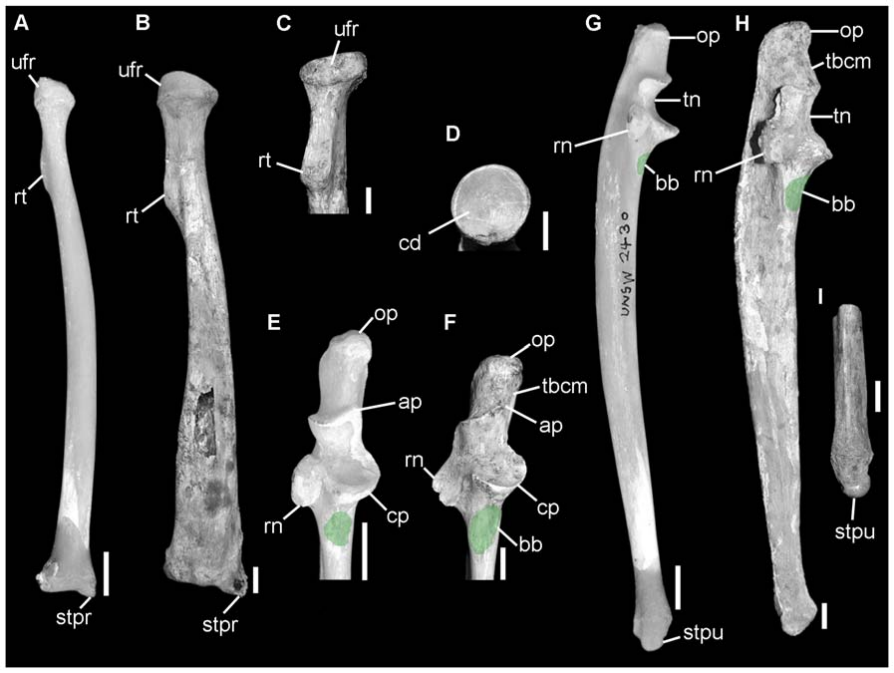
Comparison of right radius and ulna of *Phascolarctos cinereus* (A, E, G; UNSW2430) and *Nimbadon lavarackorum* (B, D, QM F40346 [mirror image]; C, QM F41097b [mirror image]; F, QM F56233; H, QM F56232; I, QM F56235a). **A–B**, radius in lateral view; **C**, proximal radius in posterior view; **D**, radius in proximal view; **E–F**, proximal ulna in anterior view; **G–H**, ulna in lateral view; **I**, distal ulna in anterior view. Abbreviations: ap, anconeal process; bb, insertion for biceps brachii and brachialis muscles; cd, capitular depression, cp, coronoid process; op, olecranon process; rn, radial notch; rt, radial tuberosity; stpr, styloid process of radius; stpu, styloid process of ulna; tbcm, insertion for triceps brachii caput mediale; tn, trochlea notch; ufr, ulnar facet of radial head. Scale bars = 1 cm.

The distal humerus and proximal radial and ulnar articulations indicate a highly mobile joint capable of significant extension of the forelimb, and supination and pronation of the antebrachium. As in *Phascolarctos*, the olecranon process is short, the anconeal process is small, and the trochlea notch is shallow and open (Figure 4E–H); features that would allow great extension of the forearm, without restricting movement to the parasagittal plane. The olecranon process is robust with deep scars on its proximal, medial and lateral surfaces for the insertion of the triceps muscles. An extended area of insertion for M. triceps brachii caput mediale is evidenced by a strong ridge extending from the anconeal process to the medial ulna (Figure 4F). The radial head is circular (contra the ovate condition in other taxa studied) with a gently concave capitular depression (Figure 4D) that would allow significant rotation about the ball-like capitulum of the distal humerus (Figure 3B). The radial notch of the ulna (Figure 4F) is flattened and laterally expanded and the corresponding articular facet on the radius is extensive (Figure 4C), encircling 270° of the radial head, which suggests significant rotary ability of the manus.

#### Radius and Ulna (Figure 4)

The *Nimbadon* ulna closely resembles that of *Phascolarctos* in being slender, relatively elongate, and mediolaterally flattened. The radius is moderately elongate, proportionately longer than *Vombatus* yet shorter relative to *Phascolarctos* and *Trichosurus*. Both the radius and ulna are proportionately and absolutely more elongate than that of *Ngapakaldia tedfordi*, a similar sized animal based on skull length. The forearm flexors (M. biceps brachii and M. brachialis) were well developed as evidenced by the broad radial tuberosity of the proximal radius (Figure 4C) and extensive insertion areas at the base of the ulnar trochlea notch (Figure 4F). The medial face of the ulna is deeply concave proximally as it is in *Vombatus* (contra *Phascolarctos*) suggesting a well developed M. flexor digitorum profundus. A broad area for the origin of M. flexor carpi ulnaris medially and extensors indicis and pollicis longus laterally, occupies the posterior surface of the ulna. Extensive flattened facets on the distal medial ulna and posterior radius for the pronator quadratus indicate a strong connection between the radius and ulna and the capacity for pronation of the manus and antebrachium.

#### Wrist joint

**Figure 5:**
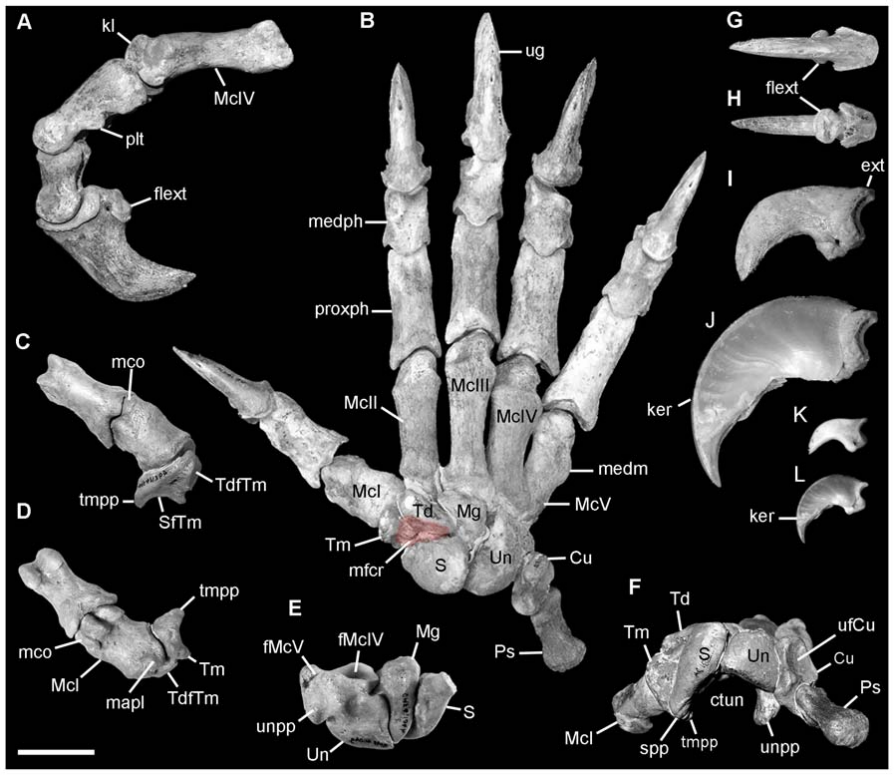
*Nimbadon lavarackorum* right manus elements. **A**, digit IV in lateral view (QM F56235); **B**, composite right manus dorsal view (specimens QM F56235, QM F56236, QM F56237); **C–D**, digit I (QM F41176, left mirror image) in **C**, dorsal view and **D**, ventral view; **E**, proximal carpal row in ventral (palmar) view (QM F41104, left scaphoid, magnum, unciform; mirror image); **F**, carpus, proximal view; **G–J**, ungual QM F56238 in **G**, dorsal view, **H**, ventral view, **I**, lateral view, **J**, lateral view with scaled *Phascolarctos cinereus* keratin sheath; **K–L**, *P. cinereus* ungual (UNSW2430) in **K**, lateral view, and **L**, lateral view with keratin sheath. Abbreviations: Cu, cuneiform; ctun, carpal tunnel; ext, extensor tubercle; flext, flexor tubercle; fMcIV, McIV facet of unciform; fMcV, McV facet of unciform; ker, keratin sheath; kl, keel; mapl, insertion M. abductor pollicis longus; Mc, metacarpal; mco, medial condyle of McI; medm, insertion of M. extensor digiti minimi; medph, medial phalanx; mfcr, tendinous insertion of M. flexor carpi radialis (red); Mg, magnum; proxph, proximal phalanx; Ps, pisiform; plt, plantar tuberosity; S, scaphoid; SfTm, scaphoid facet of the trapezium; spp, scaphoid palmar process; Td, trapezoid; TdfTm, trapezoid facet of the trapezium; Tm, trapezium; tmpp, trapezium palmar process; ufcu, ulnar facet of cuneiform; ug, ungual; Un, unciform; unpp, unciform palmar process. Scale = 2 cm.

A considerable range of movement that allows significant inversion/eversion and supination/pronation of the manus is evidenced by: a spherical ulnar styloid process (Figure 4I) and a shallowly concave circular styloid fossa of the cuneiform (Figure 5F); broadly rounded and smoothly convex radial facets of the scaphoid and unciform (Figure 5B, F); and the relatively loose concavo-convex articulation between the cuneiform and unciform. The pisiform is large and elongate, with minimal ulnar contact. It projects posterolaterally from the carpus (Figure 5B), suggesting good mechanical advantage for the flexor carpi ulnaris and enhanced rotary ability of the wrist [19]. It is more similar to *Phascolarctos* and *Trichosurus* than the robust pisiforms of *Ngapakaldia* and *Vombatus*, and like *Phascolarctos* and *Trichosurus*, has minimal articulation with the ulna. Munson [11] suggested the relatively elongate and narrow pisiforms of *Phascolarctos* and *Trichosurus* (width <0.5 length) may indicate a lifestyle or body size that requires less weight to be placed on their front feet as opposed to the robust pisiforms of large bodied terrestrial vombatoids (width >0.5 length). Alternatively, it may indicate a more digitigrade stance.

#### Manus (Figure 5)

The unciform is the largest carpal with complex concavo-convex facets for metacarpal (Mc) IV and McV indicating digits IV–V were capable of a large degree of abduction and adduction. The McIV facet is larger than the McV facet (a unique feature among the diprotodontids studied) and reminiscent of *Phascolarctos* and *Trichosurus*. The McV facet is narrow, deeply saddle-shaped, and obliquely orientated, resulting in a greater lateral projection of digit V effectively increasing the spread of the digital array. The attachment area for M. extensor digiti minimi on the mid-lateral shaft of McV is well-developed (Figure 5B) and suggestive of a significant capacity for extension without loss of mobility.

Ventrally, a large, hooked palmar process of the unciform indicates the presence of a strong transverse carpal ligament (flexor retinaculum). Correspondingly large palmar processes exist on both the scaphoid and trapezium creating a deep carpal tunnel to house the flexor tendons of the manus (Figure 5F). The magnum is a mediolaterally compressed, triangular wedge that articulates with both the unciform laterally and the scaphoid medially by smoothly concave proximal facets that allow considerable rotation around this point (Figure 5E). Weisbecker and Sánchez-Villagra [20] found this ball-joint arrangement of the unciform and magnum in possums, which enhances deviational flexibility of the hand for food manipulation and grasping branches. The mid carpal row is afforded some stability by irregular, interlocking distal articulations between the unciform and magnum (Figure 5E). Further, corresponding rugose pits on the central medial face of the magnum and the lateral face of the scaphoid indicate a strong ligamentous attachment between these carpals.

The scaphoid is the second largest of the carpals. Its dorsomedial radial facet is broadly rounded and smoothly convex and similar to that of *Trichosurus*, indicating *Nimbadon* was capable of a similar range of movement at this joint. Deep trenches on the dorsal surfaces of the scaphoid, trapezium and trapezoid form a large, rugose triangular depression for the tendinous insertion of M. flexor carpi radialis (Figure 5B).

The McI facet of the trapezium sits at an angle of 90° relative to the McII facet of the trapezoid which results in a semi-opposable pollex that projects at an angle of 60° from digit II (Figure 5B). McI is short and broad compared with the other metacarpals (Figure 5B–D). It bears no articulation with McII and the McI facet of the trapezium is shallowly concavo-convex allowing considerable movement independent of the other digits. Its diaphysis is somewhat twisted and the distal metacarpo-phalangeal articular facet is asymmetrical, with a rudimentary medial condyle, a large broad, medially positioned central condyle and narrow lateral condyle (Figure 5C–D). A prominent proximomedial process indicates a well developed M. abductor pollicis longus. These features suggest that during flexion, digit I converged on the remaining digits. Unlike all other digits of the carpus, digit I lacks a medial phalanx.

Metacarpals II–IV are elongate and robust (relative to McI and McV) with well-developed rugose tubercles proximodorsally for insertion of the digital extensors. Distally, McII-V are composed of prominent well-rounded central keels that articulate with the deeply concave proximal articular facets of the proximal phalanges. Similarly, the proximal and medial phalanges have arcuate distal condyles that interlock with deeply concave proximal articular facets of the medial and distal phalanges, respectively. This construction allows significant flexion and digit curvature in the sagittal plane (Figure 5A) while at the same time bracing against lateral stresses. In McII to McV, the lateral and central condyles are more highly developed than the medial condyle, resulting in a slightly laterally divergent metacarpo-phalangeal joint. This would effectively further increase the angle between digits II–V and digit I allowing greater abduction, opposability and potentially, grasping ability. The proximal phalanges are elongate relative to the medial phalanges. Two well developed plantar tuberosities are positioned centrally on the ventral surface of the proximal phalanges (Figure 5A). These are better developed in *Nimbadon* than in any other species studied and indicate the presence of strong flexor tendons.

The distal phalanges (unguals) of *Nimbadon* are exceedingly large (averaging 4 cm in proximodistal length), strongly curved, mediolaterally compressed, and dorsoventrally deep with well developed, ventrally prominent flexor tubercles on their proximal base (Figure 5G–I). They are very similar in morphology to those of *Phascolarctos* (Figure 5K–L), albeit more robust, and in some specimens, more arcuate. In life, with the addition of a keratin sheath, claw length would increase by at least 30% following the same proportional relationships as found in *Phascolarctos*), and significantly enhance dorsoventral depth and curvature (Figure 5J).

### Functional morphology of the hindlimb

#### Femur (Figure 6A–B)

**Figure 6:**
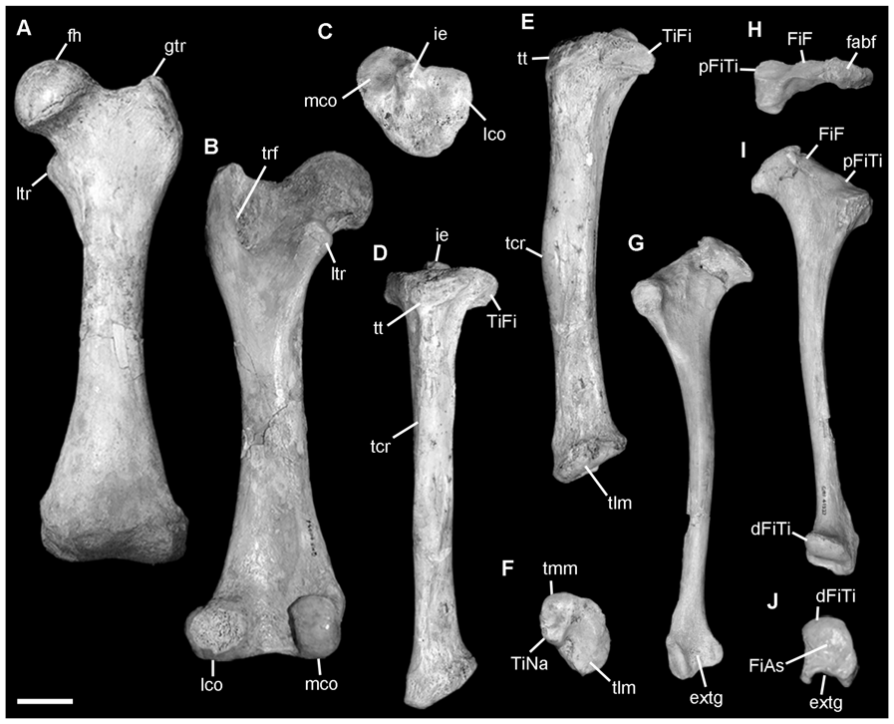
*Nimbadon lavarackorum* left hindlimb elements. **A–B** QM F41104f, femur in **A**, anterior and **B**, posterior views; **C–F**, QM F41202k, right tibia (mirror images) in **C**, proximal, **D**, anterior, **E**, lateral, and **F**, distal views; **G–J**, QM F41227, fibula in **G**, lateral, **H**, proximal, **I**, medial and **J**, distal views. Abbreviations: dFiTi, distal fibula tibial facet; extg, extensor groove; fabf, fabella facet; fh, femoral head; FiAs, fibula astragalar facet; FiF, fibula femoral facet; gtr, greater trochanter; ie, intercondylar eminence; lco, lateral condyle; ltr, lesser trochanter; mco, medial condyle; pFiTi, proximal fibula tibial facet; tcr, tibial crest; TiFi, tibia fibular facet; TiNa, tibia navicular facet; tlm, tibia lateral malleolus; tmm, tibia medial malleolus; trf, trochanteric fossa; tt, tibial tuberosity. Scale = 2 cm.

The femur is most similar in shape to that of *Ngapakaldia* and *Vombatus*, albeit proportionately shorter, however, the construction of its proximal and distal extremities are most similar to *Phascolarctos*. During climbing, the actions of abducting and externally rotating the femur assist with medially adducting the pes on the arboreal substrate [21]–[23]. In *Nimbadon*, the large globular head and less proximally extensive greater trochanter of the femur (Figure 6A) indicate enhanced mobility of the hip, particularly with regard to the abductive capacity of the M. glutei minimus and medius [21], [22]. Further, the medially (as opposed to posteriorly) positioned and distally extensive lesser trochanter seen in *Nimbadon* has been shown in arboreal mammals to enhance the abductive function of the iliacus and psoas major muscles [21]–[24] which flex, externally rotate and extend the femur during the recovery phase of locomotion [25]. The posterior face is broad and flattened (Figure 6B) providing a large insertion area for the adductors of the thigh (e.g. adductores magnus, longus, and brevis). In *Phascolarctos* these muscles are well-developed and provide the powerful adduction required for its branch hugging method of climbing. The distal articular surface is mediolaterally broad and anteroposteriorly compressed with condyles equal in height to those in *Phascolarctos* (contra all other taxa studied).

#### Tibia (Figure 6C–F)

The anterior tibial tuberosity (for M. quadriceps femoris) is large and continuous distally with an anteriorly prominent and distally extensive tibial crest (Figure 6D–E). The apex of the tibial crest defines a well developed, rugose insertion area for M. gracilis, a powerful adductor of the hip and flexor of the knee [25]. The distal tibia is morphologically most similar to *Phascolarctos* in being ovate with a lunate convex lateral malleolus and smaller medial malleolus with a large, concave navicular facet (Figure 6F) as seen in both *Phascolarctos* and *Trichosurus*.

#### Fibula (Figure 6G–J)

The fibula is robust with a narrow, slightly bowed, laterally convex, medially flat diaphysis. The proximal and distal extremities are well-developed and prominent relative to the shaft and more so in this regard than in any other taxon studied. The proximal head is anteroposteriorly elongate with a large, posteriorly extensive fabella facet (Figure 6H), indicating strong gastrocnemius and plantaris muscles (flexors of the pes). The expanded proximal head provides a large attachment area for M. peroneus longus and M. flexor digitorum fibularis. This suggests a relatively increased grasping ability [26]. The latter muscle has an extensive area of attachment on the medial face of the fibula from its head to the distal extent of the diaphysis. Distally, there is an extremely well-developed, broad and deep extensor groove developed on the external face (Figure 6G, J) for passage of the tendons that tether the peroneal muscles (longus, brevis, tertius) and M. extensor digitorum brevis.

#### Ankle joint

**Figure 7:**
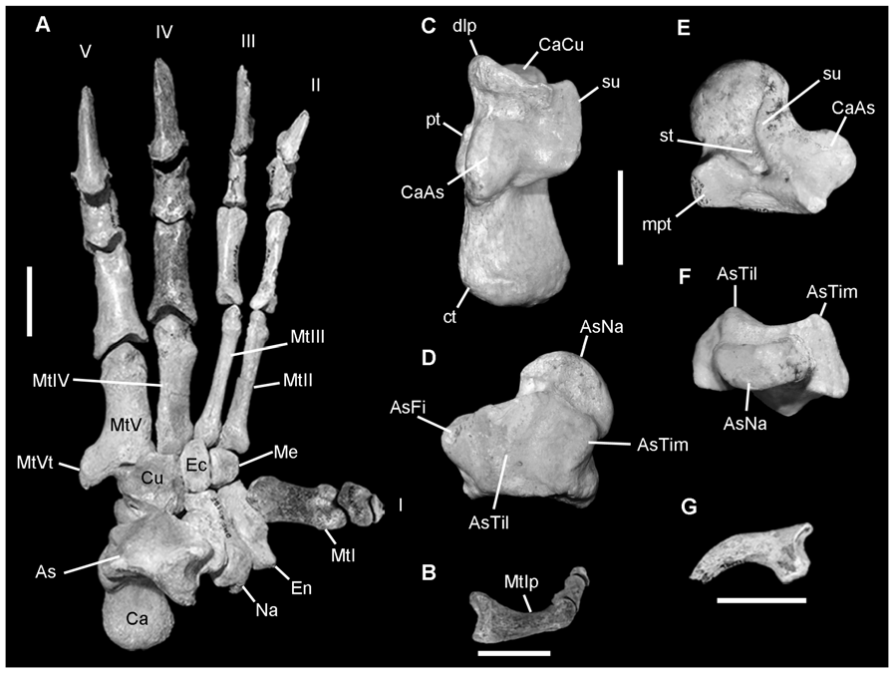
*Nimbadon lavarackorum* left pes. **A**, composite left pes, dorsal view (specimens QM F41104, QM F41108, QM F41126, QM F50523); **B**, composite hallux (QM F50523, MtI; QM F41126, proximal and medial phalanges); **C**, QM F41104n, calcaneus in dorsal view; **D–F**, QM F41104m, astragalus in **D**, dorsal view, **E**, ventral view and **F**, distal (anterior) view; **G**, QM F41210b, ungual of digit III. Abbreviations: As, astragalus; AsFi, astragalar fibular facet; AsNa, astragalar navicular facet; AsTil, astragalar tibial lateral facet; AsTim, astragalar tibial medial facet; Ca, calcaneus; CaAs, calcaneo-astragalar facet; CaCu, calcaneo-cuboid facet; ct, calcaneal tuber; Cu, cuboid; dlp, distolateral process; Ec, ectocuneiform; En, entocuneiform; Me, mesocuneiform; mpt, medial plantar tuberosity, Mt, metatarsal; MtIp, metatarsal I palmar surface; MtVt, metatarsal V tuberosity; Na, navicular; pt, plantar tubercle; st, sulcus tali; su, sustentacular; I, hallux; II, syndactylous digit II; III, syndactylous digit III; IV, digit IV; V, digit V. Scale bars = 2 cm.

Overall, the ankle joint is quite similar to that in *Phascolarctos*. In *Nimbadon* it is capable of substantial flexion and inversion/eversion as evidenced by: the shallowly concave fibula facet of the astragalus (Figure 7D) and the correspondingly smoothly convex distal end of the fibula (Figure 6I–J); and the deeply concave (both anteroposteriorly and mediolaterally) tibial facet of the astragalus (Figure 7F). The medial condyle of the astragalus (Figure 7D, 7F) has a rounded ridge that occupies the groove between the lateral and medial malleoli of the distal end of the tibia (Figure 6F) providing significant strength to the ankle joint in bracing against lateral stresses [11]. The lateral condyle of the astragalus is relatively higher than the medial condyle. This articulates with the smoothly convex and rounded lateral malleolus of the tibia. This construction is similar to that seen in *Phascolarctos* and allows plantar flexion with enhanced medial (relative to lateral) rotation and inversion of the pes. There is significant contact evident between the medial malleolus of the tibia (Figure 6F) and the dorsomedial face of the navicular such as occurs in *Phascolarctos*, *Trichosurus* and *Thylacoleo*.

#### Pes (Figure 7)

The pes is proportionately and morphologically similar to that of *Phascolarctos* although the digits of *Nimbadon* are more elongate than in any other vombatomorphian studied. Syndactylous digits II–III are exceedingly long with more gracile, less dorsoventrally deep, unguals (relative to digits IV–V) that possess a well-developed anterodorsal fissure. The hallux is medially divergent and relatively elongate, more so than any other vombatomorphian studied, and proportionately similar to both *Trichosurus* and *Phascolarctos*, albeit with a shorter proximal phalanx. As in other marsupials, the hallux is clawless and the distal-most phalanx is a short “nubbin” that directly caps the proximal phalanx. The large, oblique, concavo-convex, helical metatarsal (Mt) I facet of the entocuneiform and extensive attachment area for the tendon of the peroneus longus on the base of MtI is similar to that structure in *Phascolarctos* suggesting similar grasping capacity. MtI has an asymmetrical phalangeal facet (hypertrophied lateral condyle, smaller medial condyle) that is orientated obliquely relative to the long axis of the diaphysis. The delicate proximal phalanx shows a similar degree of torsion to MtI. This arrangement, combined with a distinctly concave MtI palmar surface (Figure 7B), would enhance opposability of the hallux by allowing it to naturally converge on the other digits during flexion [27]. The ectocuneiform and mesocuneiform taper towards the palmar surface, resulting in an arched tarsus that further enhances the grasping capacity of the pes. A deep groove is evident on the plantar surface of the cuboid for the path of the peroneus longus tendon as it traverses the tarsus to MtI. There is a well developed rugose insertion area for the tibialis anterior (an important inverter of the pes; [28]) evident on the proximo-medial plantar surface of the entocuneiform.

Significant lateral loading on the pes is indicated by: the very large proximolateral tuberosity on MtV; and the asymmetrical, proximolaterally attenuated MtV facet of the proximal phalanx. This is similar to the situation in *Ngapakaldia* and may relate to the gracile nature of the syndactylous digits II–III and opposable digit I [11]. An expanded proximolateral tuberosity of MtV also provides a large insertion area for the tendon of the peroneus brevis (a flexor and everter of the pes). Hyperextension of digits IV–V is limited by dorsal processes above the MtIV and MtV facets of the cuboid.

Concavo-convex articulations (in both the mediolateral and dorsoventral planes) at the tarsal-metatarsal interface indicate capacity for wide ranging movement at this joint. That significant weight was born by the tarsal-metatarsal joint and digits is evidenced by: the distoventrally orientated metatarsal facets of the cuneiforms and cuboid; well-developed plantar tuberosities on the tarsals and metatarsals; steeply sloping calcaneo-astragalar facet of the calcaneus; and more anteriorly projecting cubo-navicular facet. Significant flexion across the tarsus was enabled by the concavo-convex cubo-navicular facet. The calcaneus (Figure 7C) is similar in structure to that of *Phascolarctos* differing mainly in the greater robusticity in *Nimbadon* of the calcaneal tuber. The sustentacular tali projects further dorsally in *Nimbadon* and the calcaneoastragalar facet is more steeply sloping than in other taxa studied. The astragalar head is well rounded, dorsoventrally compressed and helical (Figure 7D–F) as it is in *Ngapakaldia*, *Trichosurus* and *Phascolarctos*, features that would facilitate inversion of the pes [28]. The head resembles that in *Phascolarctos* in being dorsoventrally thicker at its lateral most point, and is unique among the taxa studied in having a more semicircular, less ovate head that extends further laterally, arching through a full 180°. On the plantar surface of the astragalus is a deep groove for the tendon of the M. flexor hallucis longus (Figure 7E). Minimal contact between the astragalus and cuboid is also characteristic of arboreal taxa ([28], p.271).

## Discussion

The appendicular skeleton of *Nimbadon lavarackorum* shows striking similarities to that of the arboreal Koala (*Phascolarctos cinereus*) and arboreal/scansorial Brushtail Possum (*Trichosurus vulpecula*) in features that suggest adept climbing ability [29] and an arboreal lifestyle. These are most evident in the construction of the powerful forelimbs, the highly mobile shoulder and elbow joints, the enhanced areas of attachment for the forearm and digital flexors, and the proportionately large manus and pes, both of which display a semi-opposable digit I and very large, recurved, laterally highly-compressed ungual phalanges.

Allowing for allometric differences, *Nimbadon*'s forelimb is almost identical to that of *Phascolarctos* which suggests that it too used a trunk-hugging method of climbing. During the propulsive phase of climbing, *Phascolarctos* uses its hindlimbs to push the body upwards and its arms to grasp the trunk at higher levels [30]. The sharp, narrow but powerful claws of the manus (Figure 5) penetrate the trunk to secure each new position. Simultaneously the forearms retract to pull the body upwards while the hindlimbs, held in position by similarly structured claws, are clutched close to the body until the next propulsive thrust upwards [31]. These actions require powerful forelimb musculature as well as a highly mobile gleno-humeral joint.

In *Nimbadon*, the large rounded head of the humerus and anteriorly positioned, low humeral tuberosities indicate considerable mobility at the shoulder joint (Figure 3E–F). To compensate for the forces exerted during climbing and prevent dislocation of the shoulder, the rotator cuff muscles appear to have been extremely well-developed as evidenced by deep, rugose pits on the humeral tuberosities. The adductors and internal rotators of the humerus, the flexors of the humerus onto the scapula, as well as the major forearm, carpal and digital flexors were all powerful and would have acted synergistically during climbing to pull the body close to and maintain pressure on the tree trunk [22]–[24].

The structure of the distal humerus and proximal ulna and radius in *Nimbadon* indicate the capacity for multiaxial rotation of the elbow joint allowing a considerable range of extension, pronation and supination of the antebrachium. Great extension of the forearm is essential in climbing to facilitate reaching for new supports, whilst pronation and supination of the forearm assist with stabilising the body on an irregular three-dimensional substrate by maintaining contact of the manus with the substrate [23], [24]. The wrist was similarly flexible and capable of a high range of rotation as previously noted by Weisbecker and Archer [17] with a strongly concavo-convex scapho-radial articulation and a strongly concave cuneiform (triquetrum) facet to accommodate the well rounded styloid process of the ulna.

A striking feature of *Nimbadon* is its proportionately large manus, characterised by long slender digits, a semi-opposable pollex and exceedingly large, recurved claws (Figure 5). Combined with a relatively deep carpal tunnel (Figure 5F) and the extensive attachment areas for the carpal and digital flexors, these features suggest *Nimbadon* possessed significant grasping ability. Convergence of the pollex on the other digits of the manus would have been enhanced during flexion by torsion of the McI diaphysis and the asymmetrical morphology of the distal condyles (Figure 5C–D), both features found in the pollex of *Phascolarctos* and *Trichosurus*. Unlike *Phascolarctos*, however, *Nimbadon* did not possess a forcipate hand wherein digits I and II oppose the remaining three, effectively increasing the power of its grasp [31]. Nevertheless, the laterally projecting digit V of *Nimbadon* may have provided it with a similarly powerful grip.

Weisbecker and Archer [17] analysed phalangeal indices (PIs) and proximal and distal slenderness ratios (SRs) of the carpal phalanges for a range of diprotodontian marsupials including *Ngapakaldia* and *Nimbadon* and found that both grouped with extant arboreal possums. Interestingly, the length of digits II–IV relative to the length of the metacarpals was found to be higher in *Nimbadon* than for any other taxon studied except *Trichosurus*.

A well-developed lateral palmar process of the unciform and medial palmar processes of the scaphoid and trapezium (Figure 5F), indicate *Nimbadon* possessed a relatively deep carpal tunnel [17] which in turn is suggestive of a large flexor muscle tendon mass and strong grasping ability of the hand [23], [32]. This is further evidenced by the hyper-developed distal plantar tuberosities of the proximal phalanges (Figure 5A), enlarged proximal flexor tuberosities on the ungual phalanges (Figure 5G–I) and presence of large paired sesamoids that articulate with the distal plantar surface of the metapodials. The articular facets of the phalanges are deeply saddle-shaped, ideal for bracing against lateral stresses while their arcuate distal condyles, and those of the metacarpals and metatarsals, indicate the capacity for wide ranging flexion in the parasagittal plane (Figure 5A).

One of the most compelling arguments for climbing ability in *Nimbadon* is its exceedingly large, mediolaterally compressed and dorsoventrally deep ungual phalanges which are almost identical in shape and general morphology to those of *Phascolarctos* (Figure 5G–L). All of the digits of the manus and all but digit I of the pes possess claws of this kind. Among all marsupials studied, only scansorial and arboreal taxa possess this ungual morphology. The enlarged prominent flexor tubercle on the proximal plantar surface of the ungual indicates a strong lever arm and the capacity for powerful force to be applied to the arboreal substrate [33], [34]. As in the Koala, the claws of *Nimbadon* would be capable of deeply penetrating the tree trunk during climbing. The extensor tubercle is moderately developed and would allow moderate dorsiflexion of the unguals during walking on the ground.

The hindlimbs of *Nimbadon* appear less overtly specialised for climbing than the forelimbs and are relatively short and robust. Interestingly, however, in terms of limb proportions, *Nimbadon* displays a significantly higher intermembral index ([humerus + radius/femur + tibia] length ×100; [35]) and lower hindlimb index ([femur + tibia/vertebral column] length ×100; [35]) than any other marsupial analysed here (Table 1, Table S1, Figure S1) reflecting the extreme hindlimb reduction in this species. Comparatively high intermembral indices have been found in a range of extant clawless prosimians and are associated with increasing pedal friction on vertical surfaces during climbing [35] and/or suspensory climbing behaviours [36], wherein a shortened hindlimb functions to reduce bodymass and consequently the energy expended during suspensory behaviours [37].

**Table 1:** Comparison of forelimb and hindlimb proportions of *Nimbadon* to a range of extant and extinct marsupial taxa.

Taxon	FI	HI	IMI
*Dasyurus geoffroii*	56.6	69.4	81.6
*Sarcophilus harrisii*	68.0	70.4	96.6
*Thylacinus cynocephalus*	48.3	58.7	82.4
*Myrmecobius fasciatus*	50.3	74.0	68.0
*Isoodon obesulus*	40.0	63.3	63.2
*Phascolarctos cinereus*	64.3	66.4	96.8
*Vombatus ursinus*	50.0	59.0	84.8
*Lasiorhinus latifrons*	43.3	53.6	80.9
*Trichosurus vulpecula*	54.5	67.1	81.1
*Bettongia penicillata*	42.7	98.0	43.5
*Dendrolagus matschiei*	65.1	83.8	77.7
*Thylacoleo carnifex*	71.3	75.3	94.7
*Nimbadon lavarackorum*	59.1	49.8	118.6

Abbreviations: FI, Forelimb Index [(humerus + radius) length/vertebral column length ×100]; HI, Hindlimb Index [(femur + tibia) length/vertebral column length ×100]; IMI, Intermembral Index [(humerus + radius/femur + tibia) length ×100]. Indices (excluding those of *Nimbadon*) were calculated using measurements taken from Finch and Freedman (1988, table 1; see Table S1).

Increasing pedal friction seems an unlikely explanation for the condition in *Nimbadon*, which could have achieved significant traction through penetrating the tree trunk with its massive claws. The shortened hindlimbs in *Nimbadon* may be a further adaptation to lowering the centre of gravity during quadrupedal branch walking or when balancing on the hindlimbs while using the forelimbs to reach for new supports or food items. Nevertheless, suspensory activity may have formed part of *Nimbadon*'s positional repertoire. Certainly the powerful forelimbs, well developed rotator cuff musculature, mobile wrists and elbows, deep carpal tunnel and enhanced digital flexors and curved claws found in *Nimbadon*, would have assisted with this behaviour. Tree kangaroos, which possess many of the aforementioned morphological attributes in their forelimbs [38], have also been observed using suspensory postures [39].

The pedal morphology of *Nimbadon* is most similar to *Ngapakaldia* among diprotodontids but shares numerous features with *Phascolarctos* and *Trichosurus* that are suggestive of significant grasping ability, not least of which is the construction of the relatively elongate, semi-opposable hallux (Figure 7A). The helical, concavo-convex MtI facet of the entocuneiform allows considerable deviation of digit I both medially and ventrally. Combined with the asymmetrical distal condyles and concave ventral surface of MtI (Figure 7B), during flexion, digit I would naturally curve towards the other digits. Argot [26] suggested that in addition to an opposable hallux, other important indicators of grasping ability of the pes are the enhanced development of flexor digitorum fibularis and peroneus longus. Relative development of the former is correlated with the available space between the shafts of the tibia and fibula and is particularly well developed in arboreal species [26]. Articulation of the tibia and fibula in *Nimbadon* indicates a wide separation of the shafts which may indicate enhanced flexor digitorum fibularis muscle mass. A well developed peroneus longus is evidenced in *Nimbadon* by: an anteroposteriorly elongate head of the fibula (its point of origin); a deep groove on the lateral malleolus of the fibula (Figure 6G, 6J) and plantar surface of the cuboid (for the passage of its tendon); and the extensive insertion area on the base of MtI.

The construction of the tarsals and their pattern of articulation indicate that *Nimbadon* was capable of significant rotation and inversion of the pes. Szalay ([28], p.108) found the orientation of the head of the astragalus in marsupials can be used to predict locomotion and that transversely aligned naviculars are indicative of a “highly mobile and easily adjustable tarsus important for climbers”. In *Nimbadon* the dorsoventrally flattened semicircular head of the astragalus has an extensive mediolateral contact with the navicular; features associated with habitual inversion of the pes [28]. The *Nimbadon* astragalar-navicular facet is most similar to that of *Phascolarctos* in being relatively more dorsoventrally deep at its lateral most extent, which may in part reflect a similar inverted orientation of the pes during branch walking and the greater degree of weight placed on the lateral side of the foot. When walking along branches, the koala orients the pes laterally such that the hallux points in the direction of motion while digits II–V grasp the lateral side of the branch [31]. Other features indicating enhanced mobility at the transverse tarsal joint in *Nimbadon* include: the concave calcaneo-cuboid facet of the calcaneus and corresponding strongly rounded calcaneal facet of the cuboid; the large, convex navicular facet of the cuboid; and the limited contact between the cuboid and astragalus.

Among diprotodontids *Nimbadon* was most similar in size and postcranial morphology to the Oligocene species *Ngapakaldia tedfordi*. Munson [11] inferred a predominantly terrestrial lifestyle for *Ngapakaldia* based on ankle morphology but did suggest some climbing capacity and the possibility that *Ngapakaldia* used its well developed forelimbs and claws for uprooting bushes or stripping branches off trees. Szalay [28] also noted several arboreal correlates in the postcranial skeleton of *Ngapakaldia* and suggested that these may be plesiomorphic characters relating to the posited arboreal ancestor of diprotodontians. However, the skeleton of *Nimbadon* is even more highly modified towards an arboreal lifestyle, suggesting that these characters represent more than just a plesiomorphic carryover. Features that indicate enhanced climbing ability in *Nimbadon* relative to *Ngapakaldia* include: a proportionately larger manus and pes with proportionately longer digits; significantly larger, deeper and more recurved claws; longer forelimbs; higher intermembral index; more gracile ulna with a proportionately shorter olecranon process; and a circular as opposed to elliptical radial head.

### Palaeoecology

The question of whether *Nimbadon* was predominantly arboreal rather than arboreal/scansorial is difficult to resolve solely on the basis of the postcranial skeleton. As noted by Hildebrand ([29], p.543), the term ‘arboreal’ does not infer a mode of locomotion (as does scansorial) but rather means ‘living in trees’. Extant arboreal mammals of a similar body size to that predicted for *Nimbadon* (approx. 50–70 kg; see Text S2) are rare (however, see below). Large mammalian scansors, however, are more common. Bears, for example, are habitual climbers with several smaller species occupying rainforest habitats.

The Malayan Sun Bear *Ursus malayanus* inhabits the lowland tropical rainforests of south east Asia, Sumatra and Borneo and is the smallest (120–150 cm long, 27–65 kg) of extant bears [40]. Sun Bears are opportunistic feeders but are primarily insectivorous (termites, ants, larvae, beetles) and secondarily frugivorous and, as such, are significant seed dispersers, particularly of large seeds. Like the Koala and *Nimbadon*, Sun Bears possess large sickle-shaped claws which they utilise during climbing as well as for tearing into termite mounds, logs, and beehives to obtain insects and honey. The construction of the elbow joint in the Sun Bear shows several adaptations for digging and log tearing that, while somewhat limiting hyperextension and lateral dislocation at the elbow, allow for fast and powerful flexion and extension of the forearm in the parasagittal plane [41]. Despite having similarly large, mediolaterally compressed claws, the *Nimbadon* elbow, with its capacity for multiaxial rotation, was less capable of enduring the stresses associated with log tearing but potentially better-adapted for the complex limb movements required during climbing.

The low-crowned, lophodont dental morphology and aspects of cranial morphology indicate *Nimbadon* was primarily a browser of relatively soft, non-abrasive tree leaves [16]. Its climbing capacity would have allowed it access to multiple layers of the rainforest canopy for both food resources (to reduce competition with terrestrial kangaroos) and protection from large mammalian predators (e.g. marsupial lions). *Nimbadon* may also have opportunistically supplemented its diet with fruit as do Sun Bears. Notably, unlike other diprotodontids, *Nimbadon* possessed an anteriorly bulbous snout [16] which may indicate enhanced olfactory capacity for detecting rainforest fruits, although this awaits investigation.

The AL90 *Nimbadon* sample represents individuals ranging in age from pouch young to elderly adults [15]. Variation in cranial morphology and dimensions potentially reflecting sexual dimorphism has been identified in the sample [15], [16] yet it is relatively minimal compared with that displayed by larger diprotodontid species (e.g. *Ne. stirtoni* [7], [8]; *D. optatum* [42]). Unfortunately, sexual dimorphism in the adult postcranial skeleton of *Nimbadon* could not be discerned on the basis of the available sample.

The abundance of *Nimbadon* in the faunal assemblage of AL90 deposit suggested to Black and Hand [16] that these animals roamed in mobs. In principle there is no reason social groups of this kind common in terrestrial situations could not also have occurred within the canopy of a forest. Groups of social primates such as Orangutans can be found in close approximation, particularly when individual or adjacent trees are laden with fruit. *Nimbadon* remains the most abundant marsupial faunal component of the deposit which may reflect a collection bias with smaller, more agile animals dropping into the cave being better able to climb out [16]. Other mammalian taxa in the AL90 fauna are indicative of a diverse forested palaeoenvironment. These include: eight possum species (five pseudocheirids, two phalangerids, one burramyid); one koala (phascolarctid); four bandicoots (yaralids); three kangaroos (macropodoids); a thylacine (thylacinid); and six bats (hipposiderids) [43], [44].

The findings presented here indicate that we are only beginning to understand the range of morphological and niche diversity displayed by this ecologically important and widespread group of Australasian marsupials – the diprotodontids. *Nimbadon* is unusual among diprotodontids in having defied the general trend displayed by this group of increasing body size through time [6], [7], [9], a fact which may well reflect its arboreal habits. Despite its small size within the family, *Nimbadon* is undoubtedly the largest known marsupial herbivore to have occupied the rainforest canopies of Australia.

## Supporting Information

Text S1 **Description and comparison of** ***Nimbadon lavarackorum*** **appendicular skeleton.**
(DOCX)

Text S2 ***Nimbadon*** **body weight estimates.**
(DOCX)

Figure S1 **Plot of forelimb vs hindlimb indices for** ***Nimbadon*** **and a range of marsupial taxa.** (data from Table S1).
(DOCX)

Table S1 **Comparison of forelimb and hindlimb proportions of** ***Nimbadon*** **to a range of extant and extinct marsupials.** All measurements (means in mm) taken from Finch and Freedman (1988, table 1) except those of *Nimbadon*.
(DOCX)

## References

[1] Black K (1999) Diversity and relationships of living and extinct koalas (Phascolarctidae, Marsupialia). Aust Mamm 2: 16–17, 34–45.

[2] Black KH, Archer M, Hand SJ (2012) New Tertiary koala (Marsupialia, Phascolarctidae) from Riversleigh, Australia, with a revision of phascolarctid phylogenetics, paleoecology, and paleobiodiversity. J Vert Paleontol 32: 125–138.

[3] Black KH, Archer M, Hand SJ, Godthelp H (2012) The rise of Australian marsupials: a synopsis of biostratigraphic, phylogenetic, palaeoecologic and palaeobiogeographic understanding. In: Talent JA, editor. Earth and Life: Global Biodiversity, Extinction Intervals and Biogeographic Perturbations Through Time. Dordrecht: Springer. pp. 983–1078.

[4] Black K (2007) Maradidae: a new family of vombatomorphian marsupial from the late Oligocene of Riversleigh, northwestern Queensland. Alcheringa 31: 17–32.

[5] Stirton R, Woodburne M, Plane M (1967) A phylogeny of Tertiary Diprotodontidae and its significance in correlation. Bull Bur Min Res Geol Geophys Aust 85: 149–160.

[6] Black K (1997) Diversity and biostratigraphy of the Diprotodontoidea of Riversleigh, northwestern Queensland. Mem Qd Mus 41: 187–192.

[7] Murray P, Megirian D, Rich T, Plane M, Black K, et al (2000) Morphology, Systematics, and Evolution of the Marsupial Genus *Neohelos* Stirton (Diprotodontidae, Zygomaturinae). Museums and Art Galleries of the Northern Territory Research Report No 6: 1–141.

[8] Murray P, Megirian D, Rich T, Plane M, Vickers-Rich P (2000) *Neohelos stirtoni*, a new species of Zygomaturinae (Diprotodontidae: Marsupialia) from the mid-Tertiary of northern Australia. Mem Queen Vic Mus 105: 1–47.

[9] Black KH, Archer M, Hand SJ, Godthelp H (In press) Revision in the marsupial diprotodontid genus *Neohelos*: systematics and biostratigraphy. Acta Palaeontol Pol

[10] Price GJ, Piper KJ (2009) Gigantism of the Australian Diprotodon Owen 1838 (Marsupialia, Diprotodontoidea) through the Pleistocene. J Quat Sci 24: 1029–1038.

[11] Munson CJ (1992) Postcranial descriptions of *Ilaria* and *Ngapakaldia* (Vombatiformes, Marsupialia) and the phylogeny of the vombatiforms based on postcranial morphology. Berkeley: University of California Press. i–x: , 1–99 p.

[12] Camens A, Wells R (2010) Palaeobiology of *Euowenia grata* (Marsupialia: Diprotodontinae) and its Presence in Northern South Australia. J Mammal Evol 17: 3–19.

[13] Hand S, Archer M, Rich T, Pledge N (1993) *Nimbadon*, a new genus and three species of Tertiary zygomaturines (Marsupialia, Diprotodontidae) from northern Australia, with a reassessment of *Neohelos* . Mem Qd Mus 33: 193–210.

[14] Black K, Archer M (1997) *Silvabestius*, a new genus and two new species of primitive zygomaturines (Marsupialia, Diprotodontidae) from Riversleigh, northwestern Queensland. Mem Qd Mus 41: 181–208.

[15] Black KH, Archer M, Hand SJ, Godthelp HJ (2010) First comprehensive analysis of cranial ontogeny in a fossil marsupial-from a 15-million-year-old cave deposit in northern Australia. J Vert Paleontol 30: 993–1011.

[16] Black KH, Hand SJ (2010) First crania and assessment of species boundaries in *Nimbadon* (Marsupialia: Diprotodontidae) from the middle Miocene of Australia. Am Mus Nov 3678: 1–60.

[17] Weisbecker V, Archer M (2008) Parallel evolution of hand anatomy in kangaroos and vombatiform marsupials: functional and evolutionary implications. Palaeontol 51: 321–338.

[18] Landry SO (1958) The function of the entepicondylar foramen in mammals. Am Midland Nat 60: 100–112.

[19] Taylor ME (1974) The functional anatomy of the forelimb of some African Viverridae (Carnivora). J Morph 143: 307–335. 4837745 10.1002/jmor.1051430305

[20] Weisbecker V, Sánchez-Villagra MR (2006) Carpal evolution in diprotodontian marsupials. Zool J Linn Soc 146: 369–384.

[21] Taylor ME (1976) The functional anatomy of the hindlimb of some African Viverridae (Carnivora). J Morph 148: 227–253. 1255730 10.1002/jmor.1051480208

[22] McEvoy J (1982) Comparative myology of the pectoral and pelvic appendages of the North American porcupine (*Erethizon dorsatum*) and the prehensile-tailed porcupine (*Coendou prehensilis*). Bull Am Mus Nat Hist 173: 337–421.

[23] Argot C (2001) Functional-adaptive anatomy of the forelimb in the Didelphidae, and the paleobiology of the Paleocene marsupials *Mayulestes ferox* and *Pucadelphys andinus* . J Morph 247: 51–79. 11124686 10.1002/1097-4687(200101)247:1<51::AID-JMOR1003>3.0.CO;2-#

[24] Candela AM, Picasso MBJ (2008) Functional anatomy of the limbs of Erethizontidae (Rodentia, Caviomorpha): Indicators of locomotor behavior in Miocene porcupines. J Morph 269: 552–593. 18157864 10.1002/jmor.10606

[25] Elftman H (1929) Functional adaptations of the pelvis in marsupials. Bull Am Mus Nat Hist 58: 189–232.

[26] Argot C (2002) Functional-adaptive analysis of the hindlimb anatomy of extant marsupials and the paleobiology of the Paleocene marsupials *Mayulestes ferox* and *Pucadelphys andinus* . J Morph 253: 76–108. 11981806 10.1002/jmor.1114

[27] Argot C (2003) Functional-adaptive anatomy of the axial skeleton of some extant marsupials and the paleobiology of the Paleocene marsupials *Mayulestes ferox* and *Pucadelphys andinus* . J Morph 255: 279–300. 12520547 10.1002/jmor.10062

[28] Szalay F (1994) Evolutionary History of the Marsupials and an Analysis of Osteological Characters. Cambridge: Cambridge University Press. i–xii: , 1–463 p.

[29] Hildebrand M (1974) Analysis of vertebrate structure. New York: John Wiley and Sons.

[30] Lee AK, Carrick FN (1989) Phascolarctidae. In: Walton DW, Richardson BJ, editors. Fauna of Australia Volume 1B Mammalia. Canberra: Australian Government Publishing Service. pp. 740–754.

[31] Lee A, Martin R (1988) The Koala, A Natural History. Kensington, New South Wales: New South Wales University Press.

[32] Napier JR (1961) Prehensility and opposability in the hands of primates. Symp Zool Soc Lond 5: 115–133.

[33] Cartmill M (1979) The volar skin of primates: Its frictional characteristics and their functional significance. Am J Phys Anthropol 50: 497–510. 111555 10.1002/ajpa.1330500402

[34] MacLeod N, Rose KD (1993) Inferring locomotor behavior in Paleogene mammals via eigenshape analysis. Am J Sci 293: 300–355.

[35] Jungers WL (1978) The functional significance of skeletal allometry in *Megaladapis* in comparison to living prosimians. Am J Phys Anthropol 49: 303–314.

[36] Jungers WL, Godfrey LR, Simons EL, Chatrath PS (1997) Phalangeal curvature and positional behavior in extinct sloth lemurs (Primates, Palaeopropithecidae). Proc Nat Acad Sci 94: 11998–12001. 11038588 10.1073/pnas.94.22.11998 PMC23681

[37] Hunt KD (1991) Mechanical implications of chimpanzee positional behavior. Am J Phys Anthropol 86: 521–536. 1776659 10.1002/ajpa.1330860408

[38] Warburton NM, Harvey KJ, Prideaux GJ, O'Shea JE (2011) Functional morphology of the forelimb of living and extinct tree-kangaroos (Marsupialia: Macropodidae). J Morph 272: 1230–1244. 21630322 10.1002/jmor.10979

[39] Procter-Gray E, Ganslosser U (1986) The Individual Behaviors of Lumholtz's Tree-Kangaroo: Repertoire and Taxonomic Implications. J Mammal 67: 343–352.

[40] Stirling I, Kirshner D, Knight F (1993) Bears: Majestic creatures of the wild. New York: Rodale Press. 240 p.

[41] Salton JA, Sargis EJ (2008) Evolutionary morphology of the Tenrecoidea (Mammalia) forelimb skeleton. In: Sargis EJ, Dagosto M, editors. Mammalian Evolutionary Morphology: A Tribute to Frederick S Szalay. Dordrecht: Springer. pp. 51–71.

[42] Price GJ (2008) Taxonomy and paleobiology of the largest-ever marsupial, *Diprotodon* Owen 1838 (Diprotodontidae, Marsupialia). Zool J Linn Soc 153: 389–417.

[43] Archer M, Arena DA, Bassarova M, Beck RMD, Black K, et al (2006) Current status of species-level representation in faunas from selected fossil localities in the Riversleigh World Heritage Area, northwestern Queensland. Alcheringa 30: 1–17.

[44] Travouillon KJ, Escarguel G, Legendre S, Archer M, Hand SJ (2011) The use of MSR (Minimum Sample Richness) for sample assemblage comparisons. Paleobiology 37: 696–709.

