# Supplementary material for: Herds Overhead: Nimbadon lavarackorum (Diprotodontidae), Heavyweight Marsupial Herbivores in the Miocene Forests of Australia
Source: PLoS One. 2012 Nov 21;7(11):e48213. doi: 10.1371/journal.pone.0048213 (PMC3504027; doi:10.1371/journal.pone.0048213)
Supplement: Text S1 — Description and comparison of Nimbadon lavarackorum appendicular skeleton. (DOCX) [file pone.0048213.s001.docx]

**Text S1. Description and comparison of *Nimbadon lavarackorum* appendicular skeleton**

Skeletal comparisons were made with the arboreal Koala (*Phascolarctos* *cinereus*, AM M23550, AM M26825, AM M35240, UNSW2430, UNSW2464), the fossorial Common Wombat (*Vombatus* *ursinus*, AM M33838, AM M34032, AM S1322, AR7640), the scansorial Brushtail Possum (*Trichosurus* *vulpecula*, AM M12686, AM M34417, AM M35483, UNSWZ400) and several extinct marsupials including the diprotodontids *Ngapakaldia tedfordi* (SAM P13851 cast), *Ngapakaldia bonythoni* (QM F56235, QM F56236, QM F56237, QM F56238, QM F56239, QM F56240, SAM P13863[cast]), *Neohelos tirarensis* (QM F56242, QM F56243, QM F56244a-h, QM F50871, QM F56241a-b), *Neohelos stirtoni* (QM F56241, NMV P2329, QM F31362), *Zygomaturus trilobus* (SAM P25626, SAM P32673, SAM P75627) *Euowenia grata* (AM F4884, AM F4885, FU2671), *Diprotodon optatum* (SAM P6780, SAM P11523, SAM P5162-5164) and the Marsupial Lion, *Thylacoleo carnifex* (AM F52398, AM F106766, AM F106768, AM F106804). Comparisons were also made with the extant Malayan Sun Bear *Ursus malayanus* (AMNH 70089, AMNH 80094). Additional comparisons were made using published descriptions of skeletal remains for *Thylacoleo* (Finch and Freedman, 1988; Wells et al., 2009), *Diprotodon* (Stirling and Zietz, 1899) and *Neohelos stirtoni* (Murray et al. 2000). Reference to comparative taxa in the text is made using their generic name except for species of *Neohelos*. *Ngapakaldia* refers to *Ng. tedfordi* unless otherwise stated. Figure numbers refer to figures in the primary text.

Institutional abbreviations: AM F, Australian Museum fossil collection, Sydney; AM M and AM S, Australian Museum mammal collection, Sydney; AMNH, American Museum of Natural History mammal collection, New York; AR, collection of the Vertebrate Palaeontology Laboratory, University of New South Wales, Sydney; FU, Flinders University, palaeontology collection, Adelaide; NMV P, National Museum of Victoria palaeontology collection, Melbourne; QM F, Queensland Museum fossil collection, Brisbane; SAM P, South Australian Museum Palaeontology collection, Adelaide; UNSWZ, University of New South Wales Zoology collection, Sydney.

Description

*Humerus*

Known from three relatively complete humeri (QM F41104b, left; QM F41102e, right; QM F53644, right) and several partial humeri (QM F41097a, distal left; QM F41202a, distal left; QM F41288d, proximal left; QM F50642, distal right). QM F41102e (Figure 3B, D-G) is the most complete adult humerus, missing only some surface bone from the posterior and lateral face of the capitulum. The ectepicondylar ridge (lateral epicondylar ridge) although well preserved, appears to have been broken and healed during the individual’s lifetime with some anterior distortion and resultant rugosity. QM F41102a, a partial left humerus preserving everything distal to the deltoid crest, appears to be from the same individual as QM F41102e, and shows a smooth, dorsoventrally flattened ectepicondylar ridge and an unbroken capitulum. QM F41104b, a nearly complete left humerus is missing the proximal tip of the ectepicondylar ridge and a thin wedge of bone from the posterior surface of ectepicondylar ridge and shaft.

The overall shape and proportions of the humerus are most similar to *Phascolarctos*, being elongate with a relatively slender shaft mediolaterally and a broad distal end. Both share a well-developed and distinct linear deltopectoral crest that runs from the anteromedial border of the greater tuberosity to approximately midway along the length of the shaft where it has its greatest anterior projection in the rugose deltopectoral tuberosity. The deltopectoral crest in both *Nimbadon* and *Phascolarctos* is sharp, mediolaterally compressed with a slight medial overhang along its length and slight lateral curvature at its distal most point (Figure 3A-D). It lacks the prominent lateral overhang seen in wombats. The deltopectoral crest in *Nimbadon* differs to *Phascolarctos* in being proportionately deeper (i.e. projects further anteriorly), extends further distally along the shaft, and has a more greatly developed area for attachment of the pectoralis muscle. The scar for M. pectoralis is well developed and distinct extending along the medial side of the superior border of the deltopectoral crest and is continuous with the deltopectoral eminence. This is similar to the condition found in *Neohelos stirtoni* (Murray et al. 2000) and unlike the condition in vombatids where the deltoid tuberosity is separate and distinct from the pectoral crest. In QM F 41104b the anteroposterior depth taken at the highest point of the deltopectoral crest is 38.8mm, which occurs 102mm distal to the tip of the greater tuberosity. In QM F41202e the greatest depth of the deltopectoral crest is 40.4mm which occurs 114.9mm distal to the tip of the greater tuberosity. The insertion area for the M. deltoideus pars acromialis is a well defined triangular area with its base at the distal border of the greater tuberosity and its long pointed apex extending along the lateral superior border of the deltopectoral crest. It resembles the condition found in both *Phascolarctos* and *Trichosurus* and is unlike the broad flattened area found in *Vombatus*. A well-developed ovate muscle scar for the insertion of M. latissimus dorsi occurs on the medial surface of the shaft opposite the highest point of the deltopectoral crest. In QM F41202e it is better developed and its inferior border forms a prominent process on the posterior face of the shaft. This ovate scar is also present in *Phascolarctos* although slightly less well-developed and is comparatively weakly developed in *Trichosurus*. In *Vombatus* it is well developed though less broad than in *Nimbadon*. It is continuous proximally with a prominent linear crest for the attachment of M. teres major that runs along the posteromedial border of the shaft towards the lesser tubercle.

The ectepicondylar ridge (supracondylar ridge, lateral epicondylar crest) is more laterally extensive than in either *Phascolarctos*, *Vombatus* or *Trichosurus* and suggests a well-developed M. brachioradialis and M. extensor carpi radialis. It terminates in a well-developed medially hooked process. This hook is rarely preserved in the humeri available for study but well preserved in QM F41102e (Figure 3B). In QM F41202e, the ectepicondylar ridge extends 91.4mm from the proximal extremity which is approx 42% of the length of the humerus (compared with 32% in *Trichosurus*, 37% in *Phascolarctos* and 45% in *Vombatus*). The anterior surface of the ectepicondylar ridge is shallowly concave proximally and laterally where M. brachioradialis and M. extensor carpi radialis originate, becoming convex distally towards the trochlea. A short, shallow, narrow coronoid fossa is located on this convex surface approximately 13mm proximal to the trochlea in QM F41202e. It is similarly developed in QM F41104b yet barely visible in QM F41202a and QM F41097a. The development of the coronoid fossa resembles that in *Trichosurus* and *Phascolarctos* and contrasts to the relatively deep coronoid fossa of *Vombatus* wherein it is situated above the capitulum.

The distal articular surfaces are broad (approx. 34% the length of the humerus) and similarly proportioned to that of *Phascolarctos* and *Trichosurus*. As in these taxa, the articular surface of the trochlea is narrower, flatter and less distally extensive than the capitulum. Laterally, the capitulum is large, round and ball-like. In contrast, in *Vombatus* the trochlea is broader and more distally extensive than the capitulum. The lateral epicondyle is rugose and pitted and less prominent than in *Vombatus* yet similarly developed to that of *Phascolarctos* and *Trichosurus*. As in *Phascolarctos*, the posterolateral and lateral border of the capitulum is well defined as a diagonal (posteromedially-anterolaterally) ridge running cross the posterior and proximal articular face of the humerus. In *Vombatus* this ridge extends in the opposite direction (anteromedially and posterolaterally). The trochlea and capitulum are separated by a relatively shallow groove. Medially, a broad, well developed medial epicondyle is heavily pitted from the origin of the M. flexores digitorum profundus, superficialis and carpi radialis. It is similar in proportion to *Ngapakaldia*, slightly more laterally extensive than in *Phascolarctos* yet not as well developed as in *Vombatus*. Extending proximally and laterally from the medial epicondyle is a broad, thick entepicondylar bridge which traverses a large epicondylar (supracondylar) foramen (through which the median nerve and brachial artery pass. The entepicondylar bridge in *Nimbadon* is much broader mediolaterally than in *Vombatus* and the epicondylar foramen resembles that of *Phascolarctos* in being rounder and less ovate. A well-developed foramen is situated at the proximomedial border of the entepicondylar bridge.

Proximally, the humerus is dominated by a broad round head bounded anterolaterally by a well-developed greater tuberosity and anteromedially by a well-developed lower (i.e. more distally positioned) lesser tuberosity (Figure 3E-F). Neither tuberosities project beyond the level of the head. The proportions and positioning of the major features of the proximal humerus are very similar to that of *Phascolarctos*, and contrast to the proportionately larger and more anterolaterally extensive greater tuberosity of *Vombatus*. In *Nimbadon* the greater and lesser tuberosities are separated by a shallow intertubercular sulcus. In QM F41202e the lesser tuberosity is a broad convex ridge 23.5mm in length (proximodistally) x 12.7mm wide (anteroposteriorly) that runs distally and posteromedially from the anteromedial corner of the head. It is bordered by ridges on its anterior and posterior face with a shallow sulcus running along its length for the insertion of M. subscapularis. The muscle scar associated with the subscapularis appears better developed and more distinct than in any of the other taxa examined. In QM F41104b the ridges are less well defined, the surface is more rugose and it is longer (extending further distally) and narrower (29.8mm x 11.6mm).The greater tuberosity is dominated by deep sulci on both its proximal and lateral surfaces for the insertion of M. supraspinatus and M. infraspinatus, respectively.

The posterior face of the humerus is relatively flat as in *Phascolarctos* and contra to the rounded shaft found in both *Trichosurus* and *Vombatus*. The olecranon fossa in most *Nimbadon* humeri (positioned proximal to the trochlea) is a relatively shallow, oval depression with vague boundaries similar to that seen in *Phascolarctos* and *Trichosurus*, yet contrasts with the deep, broad fossa with distinct boundaries perforated by a small foramen found in *Vombatus*. In one *Nimbadon* specimen (QM F50642) the olecranon fossa is deep as in *Vombatus*.

# *Ulna*

Known from two relatively complete adult specimens: QM F56232, a right ulna missing the bulk of the styloid process (particularly the medial portion) and the lateral border of the lateral sigmoid cavity (Figure 4H); QM F41128, a left ulna which is fractured along the shaft and missing the proximomedial section of the olecranon process, the medial border of the radial facet and sections of the distal shaft. Partial ulnae include: QM F56233, proximal right ulna preserving 48mm of the shaft below the coronoid process (Figure 4F); QM F41202f, a proximal ulna preserving everything proximal to the coronoid process except the lateral tip of the radial facet; QM F50458 (left proximal); QM F50465 (right distal); and QM F50483 (left proximal).

The *Nimbadon* ulna is long, slender and mediolaterally flattened, reminiscent of *Phascolarctos*. It is proportionately and absolutely more elongate than that of *Ngapakaldia* and less robust both mediolaterally and anteroposteriorly. Unlike *Ngapakaldia* and *Vombatus*, the shaft does not curve medially but has a slight lateral curvature towards the distal end. This is also found in some *Phascolarctos* but may be subject to variation. The olecranon process is in-line with the shaft, deflecting only slightly medially at its tip as is the case in *Phascolarctos* and *Trichosurus* and unlike *Vombatus* wherein it is strongly deflected medially. A strong linear crest extends dorsomedially from the medial most point of the anconeal process to the anteromedial border of the olecranon process and may represent an extended site of insertion for M. triceps brachii caput mediale. This crest is not developed in any of the other taxa under study except some *Vombatus* ulnae. Medially, it delimits the well-developed fossa/sulcus for M. flexor digitorum profundus. The olecranon process is similarly short as in *Phascolarctos* and *Trichosurus* and contra that of *Vombatus*. Its superomedial surface is dominated by a rugose tuberosity that extends posteriorly and slightly laterally, fading down the posterolateral face of the olecranon process at the level of the anconeal process.

The anconeal process is small, projecting to a similar degree as in *Trichosurus* and *Phascolarctos* and unlike the well-developed process of *Vombatus*. The radial facet is broadly rounded and flat as it is in *Phascolarctos*. The trochlea notch (semilunar notch) is shallowly concave as it is in *Phascolarctos*, with a well rounded medial and distal border that articulates with the round trochlea of the humerus. Approximately 15mm distal to the distal base of the trochlea notch is a rugose, ovate (13 x 7.5 mm) muscle scar for the insertion of the M. biceps brachii. It is relatively shallow compared with the pit evident in *Trichosurus* and *Vombatus* yet similarly developed to *Phascolarctos*.

The medial face is concave proximally becoming convex distally. The medial fossa is deepest opposite the trochlear notch, suggesting a well developed M. flexor digitorum profundus proximally. The coronoid process is similarly developed to that of *Phascolarctos* and *Trichosurus* and unlike the prominent, anteromedially projecting flange of *Vombatus*. It borders a circular, shallowly concave articular fossa for the trochlea of the humerus. On the distal anteromedial face of the ulna is a well-developed flattened facet for the origin of the M. pronator quadratus. In QM F56232 it is very flat and delimited by a distinct ridge medially which gives the distal shaft a triangular or prismatic (as opposed to cylindrical) cross-section. The styloid process is spherical and resembles that of *Phascolarctos* and is similar to that of *Ngapakaldia* yet the articular surface is proportionately more rounded and less mediolaterally broad.

*Radius*

Known from five relatively complete left (QM F41097b, QM F41104c, QM F40346; QM F50547a, QM F50571a) and one right (QM F50471) adult radii. The radius in *Nimbadon* is slender and relatively elongate. It is proportionately longer than in *Ngapakaldia* but shorter than *Phascolarctos* and *Trichosurus.* In anterior profile the radius is relatively straight as it is in *Phascolarctos* and *Trichosurus* and unlike the short, medially bowed radius of *Vombatus*. The shaft proximally is cylindrical becoming deeper (anteroposteriorly) and mediolaterally flattened distally; in this respect it resembles *Vombatus* and *Ngapakaldia* more so than *Trichosurus* or *Phascolarctos*, the latter two taxa having a relatively cylindrical radial shaft along their entire length. The shaft attenuates posteriorly and is very crest-like. The anterior border is mildly convex in lateral profile, and here it resembles *Vombatus* and *Phascolarctos* more so than *Trichosurus* wherein it is relatively linear.

The radial head is unique among the taxa under study in that it is more rounded rather than ovate, although it is slightly broader mediolaterally than anteroposteriorly. The fossa for the rounded capitulum (capitular depression) is gently concave. The neck is relatively elongate, similar to *Phascolarctos* (and contra *Vombatus*). The radial tuberosity (for the attachment of the M. biceps brachii) is mediolaterally broad, only slightly narrower than the width of the radius at this point. It is flattened relative to the more prominent, medially projecting convex tuberosity of *Ngapakaldia*. The ulnar articular surface which rotates against the radial notch of the ulna is deepest (proximodistally) opposite the lateral border of the radial tuberosity.

The area of insertion of the M. pronator teres is located mid-shaft on the anterior crest of the radius and is weakly rugose. The insertion area for M. pronator quadratus on the distal posteromedial face of the radius is quite flattened and resembles *Vombatus* and *Ngapakaldia* in this respect.

The distal articular surface is sub-rectangular in shape and is similar in proportion to that of *Ngapakaldia*, *Phascolarctos* and *Trichosurus*. The medial border is relatively flat and linear whereas the lateral border is irregular in outline when viewed distally, resulting from the projection of a small tuberosity midway along its length and another at the anterolateral corner. Between these small protuberances is a generally weak groove for the M. extensor carpi radialis, however in QM F40346 it is relatively deep. A wider groove positioned more posteriorly along the lateral distal face of the radius could represent the groove for the tendon of the M. extensor pollicis longus. The radial styloid process is less prominent than in *Vombatus*, *Ngapakaldia* and *Trichosurus* and more like *Phascolarctos* in this respect. The ulnar notch (for articulation with the ulna) is a triangular, mildly convex facet on the posterodistal face of the radius. It is swollen along its posteromedial corner resulting in a small projection distally that borders the scaphoid facet. The scaphoid articular facet is gently concave posteriorly becoming more concave as it ascends the styloid process anteriorly.

*Manus* (Figure 5)

Associated elements of the manus have been found for several *Nimbadon* individuals along with several hundred isolated manual elements. The most complete specimens representing associated manual elements include: QM F41104g-i (left), QM F41173a-p (left), QM F41176a-d (left); QM F41177a-d (right), QM F41281a-h (right), QM F50511e-l (right), QM F50571b-h (left), QM F50661a-n (left), QM F56235 (right).

Unciform:— (Description based on QM F56235) The unciform is the largest of the carpals and is similar in size and shape to *Ngapakaldia* and *Trichosurus*. It articulates proximally with the radius, proximolaterally with the cuneiform, medially with both the magnum and scaphoid, distomedially (anteromedially) with McIV, and distolaterally (anterolaterally) with McV. It is compressed mediolaterally rather than dorsoventrally. The dorsal surface is roughly triangular or T-shaped, the apex of the triangle (or bottom of the 'T') being the smooth rounded proximal end. Dominated laterally by a large smooth, convex, rounded, cuneiform facet. The distoventral surface of the unciform is dominated by a large hooked process which curves back proximally (or posteroventrally). The tip of the hook (i.e., the palmar process) provides attachment for the flexor retinaculum (Johnston et al., 1958). The McIV and McV facets are relatively deep in *Nimbadon* and separated by a distinct ridge, whereas broader and shallower in *Ngapakaldia* with McV facet sloping gently into McIV facet. The McIV facet is larger than the McV facet (a unique feature among the diprotodontids studied) and reminiscent of *Phascolarctos* and *Trichosurus*. The McV facet is deeply saddle-shaped in *Nimbadon*. This narrower and deeper facet reflects the narrow proximal end of McV compared with its broad proximal end in *Ngapakaldia.* The magnum facet has a deep elongate oval trench on medial face of unciform (with a distinct cusp on its disto-ventral border), which locks onto a central prominence on the lateral face of the magnum. The facet is shallower and broader in *Ngapakaldia.* The proximolateral surface of the unciform is smooth, rounded and convex, the majority of which articulates with the radius. If viewed laterally, the most convex region lies on the midpoint of the lateral surface and is the articular facet for the cuneiform. An overhanging ridge, which runs from the dorsal surface of the unciform to the ventral palmar process, also articulates with the cuneiform.

Cuneiform:—(Description based on QM F56235) Articulates with the unciform distally and medially. The distal facet is a shallowly concave, rhomboidal-shaped facet (compared with the distinctly triangular facet in *Ngapakaldia*) that occupies approximately two thirds of the distal surface of the cuneiform. A smooth, convex, proximodorsal facet abuts an overhanging lateral ridge that extends from the dorsal surface of the unciform to the ventral palmar process. A mediolaterally directed ridge separates the proximal articular surface into a dorsal ulnar facet and a ventral pisiform facet. The ulnar facet is larger, deeper and more rounded than the shallow, sub triangular pisiform. In *Ngapakaldia* these facets are relatively equal in size (giving the distal articular face of the cuneiform its triangular shape) but deeper than those in *Nimbadon*, creating a deeper socket for the styloid process of the ulna.

Pisiform:—(Description based on QM F56235) The pisiform is relatively elongate, with a rounded, ovate ventral head and a broader triangular dorsal articular surface. It projects posterolaterally from the carpus (Figure 5B) as it does in *Phascolarctos*, *Ngapakaldia* and *Neohelos* *stirtoni*. It articulates with the cuneiform distally and the ulna medially. It is rounded in cross-section, unlike the dorsoventrally flattened pisiform of other taxa studied. In *Ngapakaldia* spp the pisiform is much more robust with a rounded ball-like ventral head twice the size of that of *Nimbadon*.

Scaphoid:—(Description based on QM F56235) The scaphoid is the second largest of the carpals In *Nimbadon*, it is teardrop shaped, as it is in *Ngapakaldia* spp, but more robust with a well-rounded ventromedial tip that articulates with a ventromedial trapezium process and a broadly rounded, smoothly convex dorsomedial surface that articulates with the radius. The ventromedial surface is concave and irregular and articulates with the magnum. The thin, distal edge of the scaphoid articulates with the trapezium (ventromedially) and trapezoid (dorsolaterally). A rough narrow groove runs the length of the scaphoid below the trapezium and trapezoid facets. This groove, along with the dorsoventral groove of the trapezium and the distomedial groove of the trapezoid, forms a roughened triangular depression on the dorsomedial face of the carpus- presumably for the attachment of the tendon of M. flexor carpi radialis. The ventrolateral surface is composed of: a rounded concave facet (at the broad end of the teardrop) that articulates with the rounded convex knob on the proximomedial base of the magnum; and an angular ridge which articulates with the ventromedial edge of the magnum and borders a deep pit that lies midway along the length of the scaphoid. When the magnum and scaphoid are articulated, the pit opposes a shallow depression on the magnum (presumably for a ligamentous attachment). The ventral external (or non-articulated) surface of the scaphoid is relatively smooth and elongate and, along with the ventral surface of the magnum, forms a flat heart-shaped surface when the McI is fully opposed. The scaphoid is similar to *Ngapakaldia* in overall morphology except in *Ngapakaldia*: the ventromedial tip is very hook-like; the ventral external surface is narrower, more crescentic and more steeply sloping; and the ventral “pit” is shallower.

Magnum:—(Description based on: QM F50455 and QM F56235). The magnum is a medium-sized, medio-laterally compressed triangular wedge that articulates with the unciform laterally and the scaphoid and trapezoid medially. It articulates distally with McIII and has a small area of contact with the lateral proximal base of McII. The McIII facet dominates the distal surface of the magnum; it is saddle-shaped (concave medially and laterally) with a dorso-ventrally concave crest on its medial third, which interlocks with the shallow central valley on the proximal base of McIII. This contrasts to the shallow, broad facet of *Ngapakaldia* (a condition midway between that seen in *Vombatus* and *Nimbadon*). A small McII facet exists as a shallow valley on the distomedial surface (i.e. distomedial face of crest which articulates with McIII). The dorsal and ventral surfaces of the magnum are much broader in *Nimbadon* than in *Vombatus*. The McIII facet of *Vombatus* is shallow, broad and flat, whereas in *Nimbadon* it is a distinct crest that slopes ventrally. The scaphoid facet is a rounded convex knob on its proximomedial base. A small rugose pit (for ligament attachment) located centrally on the medial surface of the magnum corresponds to the deep pit found on the lateral face of the scaphoid. The lateral unciform facet is dominated by a central apex, which lodges in the oval valley of the medial face of the unciform and a short depression ventral to this apex, which conforms to the large cuspate projection at the dorsoventral border of the oval valley. These structures are also present in *Ngapakaldia*. The external ventral surface of the magnum is a roughened triangular platform with a small, blunt, centrally positioned palmar process.

Trapezium:—(Description based on QM F56235) The trapezium is the most irregular in form of the carpals. It is roughly triangular as in *Ngapakaldia*, but more elongate. The McI facet is a smooth, oval depression that occupies two-thirds of its distal surface. It is shallowly concave dorsally and slightly convex ventrally to fit the concavo-convex base of McI. The scaphoid facet is composed of two small, shallow concave basins. The most ventral of these articulates with the rounded ventromedial tip of the scaphoid. The more dorsal facet sits on the narrow dorsoventral edge of the scaphoid. A sub-triangular trapezoid facet extends dorsoventrally along the trapezium’s lateral edge. The proximal face of the trapezium is dominated medially by a ridge that runs dorsoventrally and a rough, narrow groove lateral to this (for M. flexor carpi radialis).

Trapezoid:—(Description based on QM F56235) Smallest and structurally most simple of the carpals, the trapezoid is a flattened pyramidal wedge. Its flat, smooth, triangular McII facet occupies most of the distolateral surface (i.e. the base of the pyramid) except for a small concavity at its lateral most tip, where it articulates with the magnum. The trapezium facet is also a nondescript triangular facet, although smaller, occupying the medial face of the pyramid. The scaphoid facet occupies the entire proximoventral face of the pyramid. The trapezoid is similar overall to *Ngapakaldia*, except that it is larger, more triangular with three definite facets, and a more pointed/extensive proximolateral tip.

McI:—(Description based on QM F56235) McI is very broad and stout compared with the other metacarpals; greatly reduced in length such that the W/L ratio is 0.63-0.80, with a mean of 0.66 (*Ng. tedfordi* W/L= 0.6-0.7). All vombatiforms show some reduction of McI but only in *Ngapakaldia*, wombats, *Thylacoleo* and *Nimbadon*, is it reduced to such a degree. Morphology generally similar to *Ngapakaldia*, though *Nimbadon* is proportionately larger. As in *Ngapakaldia*, the proximal facet of McI is concavo-convex, dorsally convex and ventrally concave and rounded in shape and articulates with the trapezium such that it is opposable to the other digits. The distal phalangeal articular surface is asymmetrical and comprised of three condyles: a weak medial condyle that is reduced dorsally and only narrowly developed ventrally; a broad medially displaced 'central' condyle that comprises the main surface for articulation with the proximal phalanx; and a narrow lateral condyle. The diaphysis is longer laterally than medially resulting in a slight medial deviation.

McII, III, IV:—(Description based on: QM F50454a, right McII; QM F50454b, right McIII; and QM F50454c, right McIV). McII-IV are slender and elongate compared with McI and McV and are expanded proximally and distally. McII and McIV are approximately equal in length, McIII being the longest of the metacarpals. McIV the most robust of the three with a wider diaphysis at its midpoint. Distinct muscle scars for the digital extensors are evident on the dorsal surfaces of the diaphyses. The distal extremity is dome shaped and dominated dorsally by a rounded head (or central keel) that articulates with the proximal phalange (head directed slightly distolaterally). In palmar view, the distal extremities of the metacarpals are distinctly distolaterally oriented. In McII, the central keel (or condyle) is high and prominent, the lateral condyle is the largest of the three and the medial condyle exists as a small swelling at the medial base of the central condyle. Consequently, the lateral and central condyles comprise the main articular surface with the proximal phalanx. In McIII and McIV the distal articular surface is similar to McII, yet the medial condyle is larger and the central condyle is the largest of the condyles. The distal end of McIV is more robust and wider mediolaterally than those of McII-III. The proximal end of McII is mediolaterally compressed resulting in a V-shaped wedge in dorsal view. Its lateral McIII facet is well rounded. The McIII articular facet is "C"- shaped, beginning at the dorsolateral base of the shaft and curving around and back to the ventrolateral corner of the proximal end. Its trapezoid articular facet is U-shaped with broad area of contact with the trapezoid. McIII proximal end with broad concave dorsal border (as in *Ngapakaldia*); articulates with convex dorso-distal border of magnum. Medially, the McII facet of McIII is L-shaped with base of the "L" lying proximodistally (or anteroposteriorly). Laterally, the McIV facet of McIII is broad well-rounded. The magnum facet of McIII is a triangular shaped facet consisting of a ventral rounded apex and a shallow V-shaped valley, the entire surface of which articulates with the distal magnum. McIV similar proximally to McIII yet less broad, and has horseshoe-shaped unciform facet as in *Ngapakaldia*.

McV:—(Description based on QM F56235) Elongate and narrow (although not to the extent seen in *Phascolarctos* and *Trichosurus*) compared with shorter, stouter McV of *Ngapakaldia*. General shape of McV differs to McII-IV in its broad lateral flange for the insertion of M. extensor digiti minimi. This flange appears in lateral view as a well-developed crest running from the dorso-lateral base of the lateral condyle along the length of the shaft to the proximal end. The crest is less dorsoventrally concave in *Nimbadon* than *Ngapakaldia* due to the greater length of the shaft. A small foramen lies at the base of this crest on the ventrolateral face of McV at the proximal end of the shaft. The distal condyles of McV are more rounded in *Nimbadon*. The central condyle or 'median keel' is the largest and the medial condyle is larger, broader and more bulbous than the lateral condyle. The medial and lateral condyles do not flare to the degree they do in *Ngapakaldia*. Proximal end of McV broader dorsoventrally than in *Ngapakaldia* but proportionately narrower mediolaterally.

Phalanges:— (Description based on QM F56235, QM F41173, QM F50661) Proximal phalanges of digits II-V are relatively elongate (about two-thirds the length of the metacarpals), proportionately similar to *Ngapakaldia*, yet absolutely larger. On the palmar surface, two large, bilateral palmar tuberosities (which serve to hold the digital flexor tendons in place) are better developed in *Nimbadon* than in any other taxon studied. The proximal articular facet is smoothly concave mediolaterally and dorsoventrally allowing considerable dorsoventral flexion. The distal condyles are circular in medio-lateral profile and the distal facets are strongly saddle shaped and articulate with two dorsoventrally concave facets on the proximal medial phalange. The medial phalanges are short (slightly greater than half the length of the proximal phalanges) with large circular medial and lateral condyles (larger than those of the proximal phalanges) for articulation with the ungual phalanges. A large centrally positioned dorsal process projects above the proximal facet to prevent hyperextension at the phalangeal joint. The ungual phalanges are exceedingly large (averaging 4cm in proximodistal length), mediolaterally compressed dorsoventrally deep, recurved claws. They are similarly proportioned to those of *Phascolarctos* yet more robust and at times, more recurved. The articular facets of the ungual phalanges are keeled, semilunate and deeply concave, with a large and ventrally prominent flexor tubercle*.* The extent of the articular surfaces of the phalanges indicates that the digits would have been capable of a large degree of movement in the sagittal plane.

*Femur*

Description based on the relatively complete adult left femurs QM F41104f and QM F41108a and the right femurs QM F41102 and QM F50482. The femur is most similar in shape and proportion to that of *Ngapakaldia* and *Vombatus*. The diaphysis is relatively broad along its length, convex anteriorly, flattened posteriorly, and compressed anteroposteriorly. The proximal and distal ends are mediolaterally broad relative to the width of the shaft. The large globular femoral head projects further medially and less anteriorly than that of either *Phascolarctos* or *Trichosurus*, and resembles *Vombatus* in this regard. The fovea capitis for the ligamentum teres is positioned slightly posterior to centre on the femoral head. The height of the greater trochanter is reduced and does not project beyond the femoral head as it does in all other taxa studied (in some *Phascolarctos* it is level with the head, e.g. UNSW2430). The gluteal tuberosity for insertion of M. gluteus medius and M. gluteus profundus is broad and rugose. The gluteal ridge (for insertion of M. gluteus superficialis) is less distally extensive and less flange-like compared with that of *Vombatus* and more similar to that of *Ngapakaldia* and *Trichosurus*. The femoral trochanteric fossa (digital fossa) is well developed, thickly emarginated and is broad and open and similar to *Vombatus*, compared with the more slit-like fossae of *Phascolarctos* and *Trichosurus*. It is proportionately shorter than in any of the other taxa studied. The lesser trochanter is prominent and well developed as in the other taxa studied and is similar to that of *Phascolarctos* in being more posteriorly placed and in having a thicker distal crest compared with the ridge-like flange found in *Trichosurus* and *Vombatus*, yet similar to the latter in have a more distally extensive crest than *Phascolarctos*. A shallow pit for the insertion of M. psoas major is situated anterodistal to the lesser trochanter on the medial face of the diaphysis and is most similarly developed to that of *Vombatus*. A relatively weak arcuate femoral paratrochanteric crest (intertrochanteric crest) for the ischiofemoral ligament (Sargis and Szalay 2001) links the medial base of the greater trochanter with the proximolateral corner of the lesser trochanter. It is weaker than that of *Vombatus* yet more distinct than that of *Phascolarctos* and *Trichosurus*, although Sargis and Szalay (2001) found that its development in *Didelphis virginiana* varies with age, becoming more prominent in older individuals.

The postero-medial edge of the distal half of the diaphysis is rugose with a weak linear scar (for the insertion of M. adductor longus) extending to the proximal base of the medial condyle. As in all taxa studied, the distal condyles are asymmetrically developed in width yet are relatively equal in height as also seen only in *Phascolarctos*. The medial distal condyle is mediolaterally narrow and more rounded than the lateral condyle. The intercondylar notch is broad and moderately deep and proportionately more greatly separated than in any other taxon studied. A relatively deep circular pit dominates the lateral surface of the lateral condyle and a larger, semilunate but shallower pit is developed on the medial surface of the medial condyle. These are for attachment of the M. gastrocnemius and indicate *Nimbadon* may have been capable of strong flexion of the pes. Proximal to the medial pit is a small rounded tuberosity or medial epicondyle. A less prominent lateral epicondyle is situated anterior and proximal to the lateral M. gastrocnemius pit. In distal view, the medial condyle is deeper anteroposteriorly than the lateral condyle resulting from a conical anterior projection of the medial condyle, and is similar to the condition found in *Vombatus*. As with all extant diprotodontians, an ossified patella is not evident in *Nimbadon*.

*Tibia*

Known from four relatively complete adult tibias; two left (QM F41108b, QM F41110) and two right (QM F41202k, QM F50436) and several partial tibias (QM F41185, left proximal; QM F41202j, left proximal; QM F41288c, left proximal; QM F50690, right proximal; QM F50691, right proximal; QM F50692, left missing medial side; QM F50693, left proximal; QM F50842d, left distal). Description is based on QM F50436 (Figure 6C-F). In overall proportions the tibia is most similar to that of *Thylacoleo*, being less elongate than *Phascolarctos* and *Trichosurus*, yet less robust than *Vombatus*. The proximal surface is divided posteriorly into a convex lateral condyle (lateral femorotibial facet) and a larger, concave medial condyle (medial femorotibial facet), separated by a prominent, round posteromedially positioned intercondylar eminence or tubercle. The anterior portion of the proximal surface, the tibial tuberosity, is a rugose insertion area for the tendon of M. quadriceps femoris. On the lateral margin of the proximal tibia is a large, lunate fibular facet that is angled such that the distal border of this facet lies underneath the lateral condyle. When articulated, there is a wide separation between the shafts of the tibia and fibula.

The diaphysis is slightly mediolaterally compressed, convex anteriorly and concave posteriorly. It is sub-triangular in cross-section with medial, lateral and posterior surfaces. The anterior border of the tibia is dominated by a well-developed tibial crest. This crest extends from the tibial tuberosity down the anterior face, becoming more prominent approximately one third of the way down the shaft and coming to its most prominent apex approximately at the mid length of the shaft. It then curves medially, fading into the anteromedial edge of the tibia. In both *Phascolarctos* and *Trichosurus* the apex of the tibial crest is positioned higher on the shaft than in *Nimbadon* and in *Phascolarctos* bears a prominent ovate rugose muscle scar. Medial to the apex of the tibial crest is an ovate rugose depression for the attachment of M. gracilis. Proximal to this depression on the medial face of the shaft is the insertion area for the M. sartorius.

The lateral face of the tibia is dominated by a rough crest, which extends distally from the base of lateral condyle, curving anteriorly around the shaft to fade half way down the anterolateral face of the tibia. It becomes prominent again just distal of the mid-length of the shaft, then fades again into the antero-distal face of the shaft. A small nutrient foramen is positioned approximately one third of the way down the shaft on its lateral face (and is also present in *Phascolarctos*).

The distal end of the tibia (Figure 6F) is longer (anteroposteriorly) than broad (mediolaterally) and ovate in shape, most similar to *Phascolarctos*. The lateral malleolus is convex laterally (lateral astragalar-tibial facet) and convex anteromedially, becoming concave posteromedially (medial astragalartibial facet). The smaller medial malleolus exhibits a well-developed concave navicular facet as seen in both *Phascolarctos* and *Trichosurus*. The lateral and medial malleolus are separated by a short, oblique ridge. A shallow groove on the medial margin of the medial malleolus indicates the path of M. flexor tibialis. The medial astragalar-tibial facet has a deep circular pit on its posterior border (as in *Ngapakaldia* spp and all other vombatiforms) which serves for the attachment of ligaments that inhibit the amount of extension of the foot.

*Fibula*

Known from two relatively complete left adult fibula (QM F41108c, QM F41227) and several partial fibula (QM F41165a, distal; QM F50439, right distal). The T-shaped fibula is well-developed with a narrow, slightly bowed, laterally convex diaphysis and a relatively flat medial face. The proximal and distal ends are extremely well-developed and more prominent relative to the narrow shaft than in any of the taxa studied. The proximal end is anteroposteriorly elongate, and generally narrow along its length, except for a broad anterior prominence with a large, sub-ovate tibial facet that extends from the anteromedial corner to approximately half way along the length of the proximal head. The fibula articulates with the underside of the lateral condyle of the tibia. Anterior to the tibiofibula facet on the proximal fibula is a narrow, ovate femorofibular facet. The fabellar (parafibular) facet sits on the expanded posterior proximal margin which protrudes significantly from the main shaft, thus creating greater leverage for M. gastrocnemius. The fabellar facet is large and convex as it is in *Phascolarctos* but is somewhat variable in shape in *Nimbadon*, ranging from a mediolaterally narrow, elongate structure (e.g., QM F41227) to a broad, ovate structure (e.g., QM F41108c).

The fibular shaft is roundly triangular in cross-section with a flat medial surface that becomes convex distally, and convex anterolateral and posterolateral surfaces that are defined mid-shaft by a prominent ridge which appears to be the origin for M. peroneus digiti quarti. A well defined rugose ridge on the anteromedial edge extends approximately two thirds down the length of the shaft.

The distal end of the fibula is roundly convex medially (tibial and astragalarar facets) and dominated laterally by an extremely well-developed, broad and deep extensor groove for the peroneal tendons (of the peroneus longus, peroneus brevis and peroneus tertius and extensor digitorum brevis muscles; Szalay [1994]) suggesting *Nimbadon* was capable of strong eversion (pronation) of the pes.

*Pes*

Associated elements of the pes have been found for several *Nimbadon* individuals along with several hundred isolated pedal elements. The most complete specimens representing associated pedal elements include: QM F41098a-j (right), QM F41104j-y (left and right), QM F41108d-y (left), QM F41136c-l (right), QM F41137a-k (left), QM F50648a-1 (left).

Astragalus:—Description based on QM F41104m (Figure 7E-F). The astragalus of *Nimbadon* most closely resembles *Ngapakaldia* and *Phascolarctos* in overall shape and proportions. However, it differs to the latter in having a less anteriorly extensive head. As in *Ngapakaldia*, the length to width ratio is close to one. The dorsal (upper ankle joint) surface is comprised of a large concave lateral tibial facet, and a smaller, triangular fibula facet. The lateral tibial facet is bordered medially and laterally by prominent anteroposteriorly directed ridges. The lateral ridge is most prominent anterodorsally and defines the border between the lateral tibial facet and the fibular facet, whereas the medial ridge (the tibial knob of Munson [1992]) is most prominent posterodorsally and defines the dorsal border of the medial tibial facet which articulates with the medial malleolus of the tibia. The concave lateral tibial facet of *Nimbadon* is similarly developed to that of *Ngapakaldia* and *Phascolarctos* and contra the smoothly convex facet found in *Trichosurus*. Munson (1992) suggests this difference is indicative of a strong ankle joint in *Ngapakaldia* which lacks the capacity for movement found in phalangers. The medial tibial facet of *Nimbadon* is smaller, less broadly convex and less medially extensive than in *Ngapakaldia*. A relatively deep pit occupies the medial face of the astragalus, inferior to the medial tibial facet. This is also present in *Ngapakaldia* and *Trichosurus* and, to a lesser extent, *Phascolarctos*. Anteromedially, the large semicircular, dorso-ventrally flattened, head of the astragalus is dominated by the navicular facet. The navicular facet is separate from the cuboid facet, unlike the continuous semi-spherical facet seen in derived diprotodontines (Camens and Wells 2010). The navicular facet is oriented obliquely such that it is highest at its lateral margin, curving ventrally towards its medial margin. It is deeper (dorsoventrally) along its length than in *Ngapakaldia* or *Trichosurus* but not to the knob-like extent as seen in *Phascolarctos*.

On the plantar surface, the continuous concave calcaneoastragalar and convex sustentacular facets dominate the lateral half of the astragalus. A deep, crescentic sulcus tali borders these facets medially for approximately 3/4 of their length and exits onto the posterior (proximal) face of the astragalus creating a distinct notch in posterior view. This is also found in *Vombatus* and to a lesser extent in *Phascolarctos* and is contra the condition in *Ngapakaldia* wherein the sustentacular facet almost touches the medial plantar tuberosity, hence there is no sulcus between them. The medial plantar tuberosity is well-developed, prominent and rounded (knob-like), resembling more the condition in *Trichosurus* (although it is more flattened in this taxon) than either *Ngapakaldia* or *Phascolarctos*.

Calcaneus:—Description based on left (QM F41104n, Figure 7C) and right (QM F41104o) calcanea from a single individual. The *Nimbadon* calcaneus closely resembles that of *Ngapakaldia* in size, proportions and overall structure. It resembles *Phascolarctos* in overall shape, being short and wide (contra the elongate calcaneus of *Vombatus*) and differs mainly in the greater robusticity of the calcaneal tuber (tuber calcanei). As in *Ngapakaldia*, the calcaneal tuber is mediolaterally compressed, and is somewhat swollen and rugose at its posterior (proximal) end.

On its plantar surface, a thick longitudinal ridge extends towards the calcaneocuboid facet. The calcaneoastragalar facet is sinuous as in *Ngapakaldia*, obliquely aligned relative to the long axis of the calcaneus, and is concave medially (sustentaculum), becoming convex laterally. The sustentacular tali projects further dorsally in *Nimbadon* and the calcaneoastragalar facet is more steeply sloping than in other taxa studied, suggesting more weight was born over the digits than the posterior part of the tarsus; the opposite of that seen in derived diprotodontines (Camens and Wells 2010). Immediately posterior to the calcaneoastragalar facet is a shallow pit, for attachment of the calcaneoastragalar ligament. The slightly concave calcaneocuboid facet dominates the anterior (distal) face and is roundly triangular in outline as in *Ngapakaldia*, *Phascolarctos* and *Trichosurus*. It is proportionately broader dorsoventrally than in *Ngapakaldia*. The distolateral process is more prominent than in *Phascolarctos* yet reduced relative to *Ngapakaldia*, *Trichosurus* and *Vombatus*. The lateral face of the calcaneus is rugose with a well developed plantar tubercle as is the case in *Ngapakaldia*, *Phascolarctos*, *Trichosurus* and *Vombatus*.

Navicular:—Description based on the left naviculae QM F41104j and QM F41108f. The navicular of *Nimbadon* is most similar to that of *Ngapakaldia* and *Trichosurus* however it differs to the latter in having the long axis more anteroposteriorly directed rather than mediolaterally orientated, and as a result the navicular is positioned more medially relative to the astragalus in *Nimbadon*. The astragalar facet is arcuate and transversely aligned indicating a "highly mobile and easily adjustable tarsus important for climbers" (Szalay 1994; p108). The cuboid facet is shorter dorsoventrally, broader and more concave compared with that of *Ngapakaldia*, resembling that of *Trichosurus*. The convex ectocuneiform facet is similar in size to the cuboid facet and separate from the smaller convex mesocuneiform facet. The convex entocuneiform facet is ovate and positioned on the underside of the anteromedial face of the navicular such that the entocuneiform sits under the navicular. It is more elongate than in *Ngapakaldia*. The entocuneiform facet is separated from the mesocuneiform facet by a distinct elongate, shallow facet for attachment of M. abductor minimi digiti brevis. A large convex, round tibial facet occupies its posterodorsal surface. Articulation of the navicular with the tibia is also found in *Phascolarctos*, *Trichosurus* and *Thylacoleo*.

A well-developed navicular tuberosity occupies the posterior edge of the navicular. It flares medioventrally into a prominent plantar process that is significantly larger and more angular than the rounded plantar tuberosity of *Ngapakaldia* (although it is somewhat variably developed in *Nimbadon*). On the medial surface of the navicular tuberosity (at the posterior edge of the entocuneiform facet) is a deep round pit for the attachment of the tendon of M. tibialis posterior.

Cuboid:—Description based on the left cuboids QM F41104l and QM F41108g). Similar in overall shape to that of *Ngapakaldia* and also *Phascolarctos* although it is broader mediolaterally than the latter. The distal face is comprised of a large MtV facet and smaller MtIV facet which are demarcated by a slight dorsoventral ridge. Dorsally, this ridge develops into a distinct process which limits hyperextension of digits IV and V. In *Phascolarctos* the MtIV facet is larger than that of MtV and there is minimal demarcation between the two. There is minimal contact between the cuboid and astragalus in *Nimbadon*. A small convex navicular facet is located at the proximomedial face of the cuboid. It is more gently rounded compared with the ridged facet of *Ngapakaldia*, and would allow significant flexion across the tarsus. The entocuneiform articulates with the cuboid both dorsomedially and ventromedially, the dorsomedial facet being the larger of the two and is continuous with the navicular facet proximally. The calcaneocuboid articulation is also separated into two facets: a larger convex proximal facet and a smaller more flattened ventrolateral facet that extends down onto the proximal face of the plantar process; both allow significant rotation about the calcaneocuboid articulation. Ventral to the MtV facet lies a deep plantar groove for M. peroneus longus. It is similarly well-developed in *Ngapakaldia*, *Trichosurus* and *Phascolarctos*. The ventral border of the groove is defined by a relatively large plantar process.

Ectocuneiform:— Description based on the left ectocuneiforms QM F41104k and QM F41108h. The shape of the dorsal surface of the ectocuneiform mirrors that of the mesocuneiform, although it is more anteroposteriorly elongate. The lateral cuboid facet is convex and extensive. The navicular facet is extensive and concave and consists of a well-developed plantar process curving posteroventrally underneath the navicular and articulating along its length. Anteriorly, the MtIII facet is small, ovate and shallowly concave. Anterolaterally, the ectocuneiform bears a small articulation with the convex proximomedial base of MtIV.

Mesocuneiform:—Description based on the left mesocuneiforms QM F41108i and QM F50649. This is the smallest tarsal of the pes. Similar in form to that of *Ngapakaldia* and *Trichosurus* although less anteroposteriorly elongate than the latter. The lateral ectocuneiform facet is mildly concave with a linear lateral edge in dorsal view. The smaller medial entocuneiform facet is moderately concave. Anteriorly, the dorsal border of the mesocuneiform is arcuate. The MtII facet is medially concave and laterally convex and is bordered laterally by a narrow MtIII facet. A relatively elongate plantar process tapers posteroventrally projecting under the navicular. The navicular facet is small, concave and posteroventrally oriented.

Entocuneiform:—Description based on the left entocuneiforms QM F41108j and QM F50461.The entocuneiform is the largest of the cuneiform bones of the pes, is anteroposteriorly elongate, mediolaterally narrow and dorsoventrally compressed. A flat ovate navicular facet lies on its proximodorsal surface. The mesocuneiform facet is small, ovate and concave and positioned on its distolateral face. The hallucal facet for MtI is a large, twisted (almost helical), saddle shaped facet that extends half the anteroposterior (proximodistal) length of the bone from the distomedial corner. When articulated, digit I projects medially and slightly ventrally from the tarsus as it does in *Phascolarctos*. A deep pit proximal (posterior) to the hallucal facet and medial to the navicular facet may represent the attachment area for the tendon of the M. tibialis anterior; an important inverter of the pes (Szalay 1994).

Digit I:—Description based on QM F41104, QM F50523 and QM F41126. The first metatarsal (MtI) is proximally robust and elongate; similar in proportions to that of *Phascolarctos* and *Trichosurus* and larger and more elongate than *Ngapakaldia*. As in *Phascolarctos* and *Trichosurus* it tapers distally and is somewhat twisted along its long axis such that it naturally curves ventrodistally with its 'dorsal' surface facing somewhat medially. The entocuneiform facet is large, twisted and concavo-convex with a well developed central keel. The distal articular surface is also twisted, with two large condyles orientated obliquely to its long axis as in *Phascolarctos* and *Trichosurus*. The proximal phalanx of MtI is a distinct, delicate, short, dorsoventrally narrow phalanx that shows a similar degree of torsion to that of MtI. Distally, it is capped by a very small, conical phalanx. There is no ungual on digit I.

Digits II and III:— Description based on QM F41108l-m and QM F41108r-u. Digits II and III were apparently syndactylous (as in all extant diprotodontians), elongate and more slender than digits IV-V with more gracile ungual phalanges. The degree of elongation of these digits exceeds that of any other vombatomorphian studied and is most similar to those of *Phascolarctos* and *Trichosurus*. MtII is slightly shorter with a slightly thicker diaphysis than MtIII, and has a more complex concavo-convex articular facet for the mesocuneiform (relative to the simple convex ectocuneiform facet of MtIII). Both metatarsals also display strong abductor and adductor muscle attachment areas. The MtII and MtIII have a small point of articulation at their medial and lateral bases, respectively. The phalanges of MtII-III are slender and mediolaterally compressed. The unguals are more gracile, less dorsventrally deep and recurved and have a dorsal fissure at their distal tip (Figure 7A, G).

Digit IV:— Description based on QM F41098c-e, QM F41108n and QM F41104q. Digit IV is elongate (more so than digit V) and robust. The cuboid facet of MtIV is smoothly convex and proximodistally elongate. The lateral MtV facet is flattened whereas the medial MtIII facet is convex. Distally, the articular surface is hemispherical with a large central condyle (keel) and smaller medial and lateral condyles that are more distinct ventrally. The distal condyles are orientated laterally relative to the diaphysis, more so than in *Ngapakaldia*. MtIV further differs to that of *Ngapakaldia* in being more elongate, in having a more convex proximal cuboid facet and one that extends further dorsally. The phalanges of digit IV resemble those described for digits II-IV of the manus and possess bilateral plantar tuberosities for the attachment of M. flexor digitorum brevis. As in the manus, these plantar tuberosities are more strongly developed than in any other taxon studied and suggest the capacity for powerful flexion of the pes, and possibly considerable grasping ability.

Digit V:—Description based on QM F41108o, QM F41108w-x, QM F41098f-h. Digit V is distinct relative to digit IV in having a large proximolateral process on MtV that provides a large insertion area for M. peroneus brevis dorsally and exhibits a large plantar tuberosity ventrally. This process is more greatly developed and proximally extensive in *Nimbadon* than it is in *Ngapakaldia* and in both taxa is suggestive of significant lateral loading in the pes. The proximal phalanx of digit V is distinct, having an asymmetrical proximal facet with a relatively deep concavity for the middle keel of MtV, and a somewhat shallow but broad and posterolaterally attenuated facet for the enlarged lateral keel of MtV. Both of these features indicate the weight bearing nature of the lateral side of digit V. The cuboid facet of MtV is mildy convex mediolaterally and mildly concave dorsoventrally.

Comparsion of the *Nimbadon* skeleton with that of the Malayan Sun Bear (*Ursus malayanus*)

*General*

Comparisons are based on the American Museum of Natural History specimens AMNH 70089 and AMNH80094. Comparisons were made with *U. malayanus* because of potential similarities in locomotory behaviour and body size with that of *Nimbadon*. However, there were found to be few similarities in overall structure between *Nimbadon* and *U. malayanus* but this most probably relates to their divergent phylogenies rather than dissimilarity in function.

Humerus: —comparison made with AMNH70089. There are few similarities in overall morphology. The *U. malayanus* humerus lacks a well developed ectepicondylar ridge, deltopectoral crest, entepicondylar foramen, muscle scar for M. lattissimus dorsi, and the pronounced development of the greater and lesser tuberosities of the humeral head found in *Nimbadon*. The distal humerus is broader mediolaterally in *Nimbadon* and the capitulum and trochlea are more hemispherical, suggesting greater multiaxial rotation at this joint. The trochleated capitulum of the *U. malayanus* humerus is associated with fast flexion/extension of the forearm with a degree of lateral bracing (Salton and Sargis 2001). The medial epicondyle, although smaller in surface area in *Nimbadon*, projects more medially. The olecranon fossa of the *U. malayanus* humerus is deeply excavated laterally with a large anteroposteriorly convex crested process from the lateral border of the capitulum overhanging the fossa. A large convex semicircular process from the proximal head of the ulna articulates with the olecranon fossa, preventing hyperextension at the elbow and limiting rotation about the elbow joint- unlike the situation found in *Nimbadon*. The shaft is thicker and more robust in *Nimbadon*. A similar rounded development of the proximal head of the humerus occurs in both taxa although the proximal humerus is broader in *Nimbadon* resulting from the greater projection of greater and lesser tuberosities.

Radius:—comparison based on AMNH70089. *Nimbadon* radius more elongate; the shaft is flattened anterodistally for the attachment of M. pronator quadratus and is broader mediolaterally. The radial head is circular in *Nimbadon* yet obliquely subovate in the sun bear. The radial tuberosity is more greatly separated from the radial head and is positioned on the lateral face of the radius in *Nimbadon* whereas is more posteriorly orientated in the sun bear. The distal radius is more greatlymediolaterally broadened in the sun bear, the styloid process is more prominent ventrally and the articular facet is more concave.

Ulna: —comparison based on AMNH70089. *Nimbadon* ulna proportionately more elongate and mediolaterally compressed. A distinct feature of the *U. malayanus* ulna is the large convex semicircular process that projects anterolaterally from the olecranon process and articulates with the deep olecranon fossa of the humerus. The posterior border is more convex in *Nimbadon* whereas it is relatively linear in *U. malayanus*. The radial facet is concave, deeper than the very shallow facet of *Nimbadon* and proximo-distally compressed compared with ovate in *Nimbadon*. The semilunar notch is more anteriorly directed in *Nimbadon* and more concave. The styloid process of *Nimbadon* is less well-developed than in *U. malayanus* and more rounded and centrally positioned on the distal end rather than posteriorly positioned. An additional anterior articular facet is present on the distal ulna.

Manus:— (comparison is based on AMNH70089 and AMNH80094). Overall similarity in manus proportions (relative carpus/digit length) with relatively elongate slender digits and large laterally compressed claws present in both taxa. *Nimbadon* has somewhat broader, more robust digits. Comparisons are limited because the manus of both *U. malayanus* specimens are articulated with sinew still attached, thus obscuring the morphology of the individual carpals. Both taxa have large, elongate pisiforms. *Nimbadon* shows greater development of the carpal tunnel with larger plantar tuberosities on all carpals. The carpal articulation with the radius and ulna is well rounded in both taxa indicating a highly flexible wrist. The manus differs greatly in the structure of McI and the degree of opposability of digit I. In *U. malayanus* digit I is not opposable and McI resembles the other metacarpals (although it is slightly shorter) in being narrow and elongate. The distal ends of the metacarpals in *Nimbadon* are more greatly keeled and mediolaterally compressed suggesting greater capacity for flexion at this joint. The unguals are proportionately larger in *Nimbadon* and are more greatly recurved, with a much larger flexor tubercle and deeper, more arcuate articular facet.

Femur: — (comparison is based on AMNH70089 and AMNH80094). The *U. malayanus* femur is relatively and absolutely more elongate than that of *Nimbadon* with a narrow diaphysis and transversely narrower proximal and distal ends. The trochanteric fossa is smaller, shallower and thinly emarginated in *U. malayanus*. The greater trochanter is less proximally extensive, sitting well below the level of the femoral head in *U. malayanus* (contra the condition in *Nimbadon* where the tip of the greater trochanter is level with the femoral head). The lesser trochanter of *U. malayanus* is smaller, more laterally and distally positioned on the diaphysis with respect to the femoral head and does not project medially from the diaphysis as it does in the well-developed flange-like lesser trochanter of *Nimbadon*. Distally, the rotular surface is proximodistally extensive, mediolaterally narrow and more concave in *U. malayanus* with more distinctly defined condylar ridges.

Tibia:—(comparison is based on AMNH70089 and AMNH80094). The tibia of *U. malayanus* is similar in absolute length and general proportions, albeit the diaphysis in *Nimbadon* is somewhat thicker proximally and at its midline and less anteroposteriorly flattened distally. Proximally, the *U. malayanus* tibia differs mainly in the absence of a prominent rounded intercondylar eminence and the large lateral fibula facet. The anterior crest of the diaphysis of *U. malayanus* is reduced and restricted to a more proximal position whereas it is most prominent mid way along the length of the diaphysis in *Nimbadon*. The mediolaterally expanded distal articular surface of the *U. malayanus* tibia is significantly different to the anteroposteriorly expanded facet found in *Nimbadon*. The astragalar facet of *U. malayanus* is deeply concavo-convex to articulate with the well developed arcuate anteroposteriorly convex surface of the astragalus. Consequently, movement at this joint in *U. malayanus* would be limited to the sagittal plane as opposed to the greater mobility evident in *Nimbadon*.

Fibula:—comparison is based on AMNH70089. The fibula of *U. malayanus* is a narrow splint of bone with limited weight bearing function, unlike that of the well developed *Nimbadon* fibula. As such, few comparisons can be made.

Pes: — As with the manus, comparisons are limited because of the articulated, sinew covered nature of the *U. malayanus* specimens. Aside from the absence of syndactylous digits II and III in the *U. malayanus* pes, the most obvious differences to the *Nimbadon* pes are the absence of both a semi opposable digit I and the enlarged (relative to the other metatarsals ) MtV with a well developed lateral tuberosity. Because of the relatively reduced MtV the cuboid of *U. malayanus* is correspondingly smaller. The astragalus of *U. malayanus* exhibits a dorsoventrally deep navicular facet indicating greater movement in the sagittal plane compared with the mediolaterally broad, arcuate articular facet in *Nimbadon* that allows considerable mediolateral rotation. The pes is plantigrade in *U. malayanus* yet somewhat more digitigrade in *Nimbadon*.

References

Argot C (2001) Functional-adaptive anatomy of the forelimb in the didelphidae, and the paleobiology of the Paleocene marsupials *Mayulestes ferox* and *Pucadelphys andinus*. J Morph 247: 51-79.

Argot C (2003) Functional-adaptive anatomy of the axial skeleton of some extant marsupials and the paleobiology of the Paleocene marsupials *Mayulestes ferox* and *Pucadelphys andinus*. J Morph 255: 279-300.

Camens A, Wells R (2010) Palaeobiology of *Euowenia grata* (Marsupialia: Diprotodontinae) and its Presence in Northern South Australia. J Mammal Evol 17: 3-19.

Candela AM, Picasso MBJ (2008) Functional anatomy of the limbs of Erethizontidae (Rodentia, Caviomorpha): Indicators of locomotor behavior in Miocene porcupines. J Morph 269: 552-593.

Elftman H (1929) Functional adaptations of the pelvis in marsupials. Bull Am Mus Nat Hist 58: 189-232.

Finch M, Freedman L (1988) Functional-morphology of the limbs of *Thylacoleo carnifex* Owen (Thylacoleonidae, Marsupialia). Aust J Zool 36: 251-272.

Landry S.O. 1958 The function of the entepicondylar foramen in mammals. Am Mid Nat 60: 100-112.

McEvoy J. 1982 Comparative myology of the pectoral and pelvic appendages of the North American porcupine (*Erethizon dorsatum*) and the prehensile-tailed porcupine (*Coendou prehensilis*). Bull Am Mus Nat Hist 173: 337-421.

Munson CJ (1992) Postcranial descriptions of *Ilaria* and *Ngapakaldia* (Vombatiformes, Marsupialia) and the phylogeny of the vombatiforms based on postcranial morphology. Berkeley, University of California Press; i-x, 1-99 p.

Murray P, Megirian D, Rich T, Plane M, Black K, Archer M, Hand S, Vickers-Rich P (2000) Morphology, systematics, and evolution of the marsupial genus *Neohelos* Stirton (Diprotodontidae, Zygomaturinae). Museums and Art Galleries of the Northern Territory Research Report No 6, 1-141.

Sargis EJ, Szalay FS (2001) Model-based analysis of postcranial osteology of marsupials from the Palaeocene of Itaboraí (Brazil) and the phylogenetics and biogeography of Metatheria. Geodiversitas 23: 139-302.

Stirling EC, Zietz AHC (1899) Fossil remains at Lake Callabonna Part 1. Description of the manus and pes of *Diprotodon australis*, Owen. Mem Roy Soc S Aust 1: 1-40.

Szalay F (1994) Evolutionary History of the Marsupials and an Analysis of Osteological Characters. Cambridge, Cambridge University Press; i-xii, 1-463 p. 1.

Taylor ME (1976) The functional anatomy of the hindlimb of some African Viverridae (Carnivora). J Morph 148: 227-253.

Weisbecker V, Sánchez-Villagra MR (2006) Carpal evolution in diprotodontian marsupials. Zool J Linn Soc 146: 369-384.

Wells RT, Murray PF, Bourne SJ (2009) Pedal morphology of the Marsupial Lion *Thylacoleo carnifex* (Diprotodontia: Thylacoleonidae) from the Pleistocene of Australia. J Vert Paleontol 29: 1335-1340.
