# Supplementary material for: Herds Overhead: Nimbadon lavarackorum (Diprotodontidae), Heavyweight Marsupial Herbivores in the Miocene Forests of Australia
Source: PLoS One. 2012 Nov 21;7(11):e48213. doi: 10.1371/journal.pone.0048213 (PMC3504027; doi:10.1371/journal.pone.0048213)
Supplement: Text S2 — Nimbadon body weight estimates. (DOCX) [file pone.0048213.s002.docx]

**Text S2: *Nimbadon* body weight estimates**

Previous body weight estimates of 75-120 kg for *Nimbadon* were calculated by Black and Hand (2010), based on Anderson et al.'s (1985) equation (listed below), yet are believed to be inaccurate resulting from inflated C_h_ values taken at the humeral mid-shaft too close to the prominent deltopectoral crest. C_h_ values used here represent absolute minimum humeral shaft circumference values taken distal of the deltopectoral crest. In addition body weight estimates were also calculated using Myers (2001) predictive regression equations (which correlate diprotodontian cranio-dental variables with body weight).

Calculated using Anderson et al.'s (1985) equation:

log(body mass) = -1.42 + 2.89 log (C_h+f_)

where 'h' and 'f' are minimum midshaft circumference ( in mm) of humerus and femur, respectively

C_h_ = 78mm (QM F41202e)

C_f_ = 67 mm (QM 41104f); 69 mm (QM F50482); mean = 68 mm (n=2)

Body mass = **68.4 kg**

Calculated using Myers (2001) equations who found the Total Jaw Length (TJL), Upper Molar Occlusal Row Length (UMORL) and 3^rd^ Upper Premolar Width (UP3W) to be the best predictors for diprotodontian body weight estimates:

Total jaw length

log(body mass) = -2.884 + 3.426 (log x); where x = TJL

x=192. 3mm (QM F40337); Body mass = **87.3 kg**

Upper molar occlusal row length

log(body mass) = -0.567 + 3.072 (log x); where x = UMORL

x = 53.0 mm (n = 4; mean of left UMORL for adult crania QM F50470 [53.4 mm], QM F53645a [55.9 mm], QM F31541 [51.7 mm], QM F40346 [51.0 mm] ); Body mass = **53.7 kg** (range = 47.7- 63.2 kg)

Third upper premolar width

log(body mass) = 1.775 + 2.991 (log x); where x = UP3W

x = 10.4 mm (n = 28; Black and Hand 2010); Body mass = **65.6 kg** (range 9.4-12.0 mm; 48.5- 100.7 kg)

References

Anderson J, Hall-Martin A, Russell D (1985) Long-bone circumference and weight in mammals, birds and dinosaurs. J Zool Soc Lond (A) 207: 53-61.

Black KH, Hand SJ (2010) First crania and assessment of species boundaries in *Nimbadon* (Marsupialia: Diprotodontidae) from the middle Miocene of Australia. Am Mus Nov 3678: 1-60.

Myers TJ (2001) Prediction of marsupial body mass. Aust J Zool 49: 99-118.
