# Supplementary material for: Herds Overhead: Nimbadon lavarackorum (Diprotodontidae), Heavyweight Marsupial Herbivores in the Miocene Forests of Australia
Source: PLoS One. 2012 Nov 21;7(11):e48213. doi: 10.1371/journal.pone.0048213 (PMC3504027; doi:10.1371/journal.pone.0048213)
Supplement: Figure S1 — Plot of forelimb vs hindlimb indices for Nimbadon and a range of marsupial taxa. (data from Table S1). (DOCX) [file pone.0048213.s003.docx]

**Figure S1. Plot of forelimb vs hindlimb indices for *Nimbadon* and a range of marsupial taxa.** (data from Table S1)
