# Supplementary material for: Herds Overhead: Nimbadon lavarackorum (Diprotodontidae), Heavyweight Marsupial Herbivores in the Miocene Forests of Australia
Source: PLoS One. 2012 Nov 21;7(11):e48213. doi: 10.1371/journal.pone.0048213 (PMC3504027; doi:10.1371/journal.pone.0048213)
Supplement: Table S1 — Comparison of forelimb and hindlimb proportions of Nimbadon to a range of extant and extinct marsupials. All measurements (means in mm) taken from Finch and Freedman (1988, table 1) except those of Nimbadon. (DOCX) [file pone.0048213.s004.docx]

**Table S1**. **Comparison of forelimb and hindlimb proportions of *Nimbadon* to a range of extant and extinct marsupials.** All measurements (means in mm) taken from Finch and Freedman (1988, table 1) except those of *Nimbadon*.

| Taxon | N | Humerus | Radius | FI | Femur | Tibia | HI | VC | IMI |
| --- | --- | --- | --- | --- | --- | --- | --- | --- | --- |
| *Dasyurus geoffroii* | 15 | 57.3 | 56.9 | 56.6 | 66.8 | 73.2 | 69.4 | 201.6 | 81.6 |
| *Sarcophilus harrisii* | 4 | 97.0 | 96.7 | 68.0 | 100.3 | 100.2 | 70.4 | 284.7 | 96.6 |
| *Thylacinus cynocephalus* | 1 | 159.9 | 152.0 | 48.3 | 194.5 | 184.1 | 58.7 | 644.6 | 82.4 |
| *Myrmecobius fasciatus* | 5 | 33.0 | 32.6 | 50.3 | 45.5 | 50.9 | 74.0 | 130.3 | 68.0 |
| *Isoodon obesulus* | 20 | 45.8 | 37.3 | 40.0 | 65.2 | 66.3 | 63.3 | 207.6 | 63.2 |
| *Phascolarctos cinereus* | 1 | 105.7 | 117.2 | 64.3 | 128.6 | 101.6 | 66.4 | 346.6 | 96.8 |
| *Vombatus ursinus* | 1 | 112.6 | 105.5 | 50.0 | 143.3 | 113.9 | 59.0 | 436.0 | 84.8 |
| *Lasiorhinus latifrons* | 1 | 104.5 | 99.1 | 43.3 | 135.1 | 116.7 | 53.6 | 470.2 | 80.9 |
| *Trichosurus vulpecula* | 20 | 59.2 | 66.4 | 54.5 | 77.5 | 77.3 | 67.1 | 230.6 | 81.1 |
| *Bettongia penicillata* | 5 | 35.3 | 44.8 | 42.7 | 78.1 | 106.0 | 98.0 | 187.8 | 43.5 |
| *Dendrolagus matschiei* | 2 | 101.4 | 96.6 | 65.1 | 125.5 | 129.2 | 83.8 | 304.0 | 77.7 |
| *Thylacoleo carnifex* | 1 | 224.0 | 258.0 | 71.3 | 279.0 | 230.0 | 75.3 | 676.0 | 94.7 |
| *Nimbadon lavarackorum* |  | 210.5* | 194.4^#^ | 59.1 | 183.4^‡^ | 158.0^$^ | 49.8 | 685.0^ | 118.6 |

VC = vertebral column length (axis to last lumbar)

Forelimb index (FI): (humerus + radius)/VC X 100

Hindlimb index (HI): (femur + tibia)/VC X 100

Intermembral Index (IMI): (humerus + radius / femur + tibia) length X 100

**Nimbadon* humerus mean length calculated from 3 individuals: QM F41104b, QM F41202e, QM F53644

^#^*Nimbadon* radius mean length calculated from 6 individuals: QM F41104c, QM F50571a, QM F41097b, QM F50547a, QM F50471, QM F40346

^‡^ *Nimbadon* femur mean length calculated from 4 individuals: QM F41102, QM F41104f, QM F41108a, QM F50482

^$^*Nimbadon* tibia mean length calculated from 3 individuals: QM F41108b, QM F41110, QM F50436

^ *Nimbadon* vertebral column length determined from composite of 5 individuals: C1-C5 QM F41130; C6 QM F50712; C7-T1 QM F50478; T2-T10 QM F 41202; T11-L6 QM F50714

Reference

Finch M, Freedman L (1988) Functional-morphology of the limbs of *Thylacoleo carnifex* Owen (Thylacoleonidae, Marsupialia). Aust J Zool 36: 251-272.
